# Screening of co-pathogenic genes of non-alcoholic fatty liver disease and hepatocellular carcinoma

**DOI:** 10.3389/fonc.2022.911808

**Published:** 2022-08-11

**Authors:** Ting Chen, Siwen Zhang, Dongmei Zhou, Peipei Lu, Xianglai Mo, Rashi Tamrakar, Xi Yang

**Affiliations:** ^1^ Department of Endocrinology, First Affiliated Hospital, Guangxi Medical University, Nanning, China; ^2^ Department of Gastrointestinal Surgery, First Affiliated Hospital, Guangxi Medical University, Nanning, China; ^3^ Department of Geriatric Endocrinology and Metabolism, First Affiliated Hospital, Guangxi Medical University, Nanning, China; ^4^ Guangxi Key Laboratory of Precision Medicine in Cardio-Cerebrovascular Diseases Control and Prevention, First Affiliated Hospital, Guangxi Medical University, Nanning, China; ^5^ Guangxi Clinical Research Center for Cardio-Cerebrovascular Diseases, Nanning, China

**Keywords:** ABCC5, bioinformatics, hepatocellular carcinoma, nonalcoholic fatty liver disease, TUBG1

## Abstract

**Background:**

Non-alcoholic fatty liver disease (NAFLD) is a risk factor for hepatocellular carcinoma (HCC). However, its carcinogenic mechanism is still unclear, looking for both diseases’ transcriptome levels, the same changes as we are looking for NAFLD may provide a potential mechanism of action of HCC. Thus, our study aimed to discover the coexisting pathogenic genes of NAFLD and HCC.

**Methods:**

We performed a variance analysis with public data for both diseases. At the same time, weighted gene correlation network analysis (WGCNA) was used to find highly correlated gene modules in both diseases. The darkturquoise gene module was found to be highly correlated with both diseases. Based on the diagnosis related module genes and the differential genes of the two diseases, we constructed diagnostic and prognostic models by logistic regression, univariate Cox regression, and LASSO regression. Public datasets verified the results. Meanwhile, we built a competing endogenous RNA (ceRNA) network based on the model genes and explored the related pathways and immune correlation involved in the two diseases by using Gene Ontology, Kyoto Encyclopedia of Genes and Genomes, and gene set enrichment analyses. Immunohistochemistry was used to verify the different expression of ABCC5 and TUBG1 among the normal liver, NAFLD, and HCC tissues. Sodium palmitate/sodium oleate was used to establish high-fat cell models, and Real Time Quantitative Polymerase Chain Reaction (RT-qPCR) was used to verify the messenger RNA (mRNA) expression of ABCC5 in lipidization cells.

**Results:**

A total of 26 upregulated genes and 87 downregulated genes were found using limma package identification analysis. According to WGCNA, the darkturquoise gene module was highly correlated with the prognosis of both diseases. The coexisting genes acquired by the two groups were only three central genes, that is, ABCC5, DHODH and TUBG1. The results indicated that the diagnostic and prognostic models constructed by ABCC5 and TUBG1 genes had high accuracy in both diseases. The results of immunohistochemistry showed that ABCC5 and TUBG1 were significantly overexpressed in NAFLD and HCC tissues compared with normal liver tissues. The Oil Red O staining and triglyceride identified the successful construction of HepG2 and LO2 high-fat models using PA/OA. The results of RT-qPCR showed that the lipidization of LO2 and HepG2 increased the mRNA expression of ABCC5.

**Conclusions:**

The gene model constructed by ABCC5 and TUBG1 has high sensibility and veracity in the diagnosis of NAFLD as well as the diagnosis and prognosis of HCC. ABCC5 and TUBG1 may play an important role in the development of NAFLD to HCC. In addition, lipidization could upregulate the mRNA expression of ABCC5 in HCC.

## Introduction

Cancers like hepatocellular carcinoma (HCC) have one of the highest mortalities in the world. Pathological metabolic diseases are the primary causes of HCC, such as hepatitis C or B and alcoholic and non-alcoholic fatty liver disease (NAFLD) (1). Due to the rapid increase in obesity and NAFLD patients, the prevalence of NAFLD-related HCC has increased accordingly (2). As the harmful effects of NAFLD can ultimately cause cirrhosis and even cancer, the prevalence of HCC has also started to rise suddenly. Based on some research findings (3), NAFLD can act synergistically with other HCC risk factors to accelerate cancer development. Over the past decade, an analysis of US health insurance databases found (4), according to the report, that 59% of HCC patients enrolled in medical plans are believed to have NAFLD as their primary cause. In a meta-analysis by Younossi et al. (5), all studies from 1989 to 2015 involving NAFLD were screened and estimated to determine point estimates [95% confidence intervals (CIs)] of HCC incidence, mortality, and incidence rates using a random-effects model. In NAFLD patients, HCC incidence was found to be 0.44/1,000 person-years (range: 0.29–0.66). A retrospective cohort study (6) evaluated the cancer risk in patients with NAFLD; it is also required to follow up the successive prevalence of HCC. Tumor biomarkers can assist medical researchers with early screening, diagnosis, treatment evaluation, recurrence, and prognosis prediction of tumors. With the prevalence of HCC and the estimated continued increase in this tumor (7), it is suggested that early monitoring and screening of tumor markers can reduce the mortality rate. In fact, in most patients, even after a complete resection or ablation of HCC tumors, the remaining carcinogenic tissue will progress to incurable terminal disease (8), thus, early detection and prevention of HCC development are considered to be the most effective strategies worldwide for improving patient outcomes. Up to now, the exact pathogenesis of NAFLD transition to HCC has not been explained. Identifying new marker genes can be used to predict risk and target treatment for individual patients. Hence, the prediction of candidate genes based on NAFLD-HCC pathogenesis is also an option. Based on bioinformatics analysis, earlier studies have established that telomerase reverse transcriptase (hTERT) (9), hypoxia-inducible transcription factor-2a (HIF-2α) (10), S100 calcium-binding protein A11(S100A11) (11), squalene epoxidase (SQLE) (12), and deoxycytidine kinase (DCK) (13) were associated with NAFLD to HCC progression and prognosis and could act as a related marker. Therefore, in an era of precision medicine, the diagnostic markers of NAFLD progressing to HCC need to be explored further.

In the human genome, the ATP-binding cassette (ABC) transporter is one of the largest protein families. More than 48 genes encoding human ABC transporters have been identified and sequenced, and the largest member of the family is multidrug resistance-associated protein (MRP), including MRP1 (ABCC1), MRP5 (ABCC5), and MRP6 (ABCC6) (14). In addition to its role in chemotherapy-resistant tumors, as one of the major classes of membrane ATPase, it is also implicated in the treatment failure of HCC with a multidrug-resistant phenotype (15). Studies in NAFLD have shown that several ABC transporter family members, such as ABCA1 and ABCC5, are closely related to the progression of NAFLD (16).

γ-Tubulin (TUBG1) is a member of the human tubulin family. There are five known tubulin subtypes in the human tubulin family based on the c-terminal region with the greatest variation: α-tubulin, β-tubulin, γ-tubulin, δ-tubulin, and ϵ-tubulin. Complex cortical malformations related to tubulin gene mutations, also known as tubulin diseases, are a heterogeneous group of diseases with a wide range of clinical severity (14). A family of GTP enzymes called tubulin is highly enriched in microtubules and centrosomes.

This study aimed to identify candidate biomarkers for predicting NAFLD and HCC diagnoses by using the full public repository for Gene Expression Omnibus (GEO) and The Cancer Genome Atlas (TCGA) databases. We screened differentially expressed genes (DEGs) from public datasets of NAFLD and HCC and also screened overlapping genes between DEGs and the constructed weighted gene correlation network analysis (WGCNA); constructed NAFLD and HCC models, functional and pathway enrichment analyses, established ceRNA network, and protein–protein Interaction (PPI) network, respectively, and finally identified the hub gene. Therefore, this study hopes to provide new candidate diagnostic markers during the progression of NAFLD to HCC, offer the best screening strategy for high-risk patients, and advance the accuracy of HCC prevention.

## Materials and methods

### Data download and processing

Through the GEOquery package (17), NAFLD datasets GSE48452, GSE89632, and GSE37031 were downloaded from the GEO database. The GSE48452 dataset is from *Homo sapiens*, and the data platform is GPL11532, which has 73 samples in total, including 14 normal liver tissue control samples, 27 liver tissue samples from obese patients, and 32 NAFLD tissue samples (18).This study included 14 normal liver tissue control samples and 32 NAFLD tissue samples from the dataset. The GSE89632 dataset is from *Homo sapiens*, and the data platform is GPL14951, which contains 63 samples, including 24 normal liver tissue control samples and 39 NAFLD tissue samples. This study included all the samples (19). The GSE37031 dataset is from *Homo sapiens*, and the data platform is GPL14877, containing 15 samples, including 7 normal liver tissue control samples and 8 NAFLD tissue samples. In this study, all samples were included as a validation set (20). GSE48452 and GSE89632 datasets are combined, and batches are removed through the SVA package (21). The data were normalized and standardized through the limma package (22).

Through the TCGAbiolinks package (23), the hepatoma dataset was downloaded from the TCGA database, TCGA-LIHC (Liver Hepatocellular Carcinoma, n=445 cases). The data type Count was selected and converted to the TPM format. HCC data were taken from the International Cancer Genome Consortium database, and TCGA-LIHC (n = 419 cases) was used as a validation set.

### Model building and validation

For the construction of diagnostic models, the minimization of absolute contraction and selection operator LASSO regression are commonly used machine learning algorithms. In curve fitting, regularization is used to solve the overfitting and improve model accuracy. The glmnet package (24) was used to construct the model, and the parameter was set as: seed (2), family = “ binomial “.

The prognostic model of HCC patients was developed using univariate and multivariate COX regression.

### Variance analysis

The limma package was used for the gene difference analysis of GEO chip data in the normal group and disease group, and R package DESeq2 was used for the gene difference analysis of the normal group and disease group (25). LogFC >0.5 and the adjp value <0.01 were fixed as the threshold of differential genes. LogFC >0.5 was considered to be upregulated in the high-risk group, while logFC <0.5 was downregulated. The outcomes of difference analysis were represented by R package pheatmap heatmap and GGplot2 volcano map (26).

### Analysis of enrichment (gene ontology/kyoto encyclopedia of genes and genomes/gene set enrichment analysis/GSVA/ssGSEA)

Gene Ontology (GO) is a common analysis for large-scale functional enrichment studies, such as biological processes (BPs), molecular functions (MFs), and cellular component (CC). The Kyoto Encyclopedia of Genes and Genomes (KEGG) is a widely used database keeping information about genomes, biological pathways, diseases, and drugs. The Cluster Profiler R software package was used to analyze the GO annotation and KEGG pathway enrichment analysis of differential genes, and the critical value of False discovery rate(FDR) < 0.05 was considered statistically significant (27).

The gene set enrichment analysis (GSEA) method analyzes whether a particular gene set is statistically different between two biological states and is commonly used to estimate changes in the pathway and biological process activities in expression dataset samples (27). For GSEA analysis, the “msigdb. v7.0. Symbols” gene set was downloaded from the MSigDB database.

Furthermore, the enrichment fractions of related pathways in the MSigDB database were calculated according to the gene expression matrix of each sample using the Gene Set Variation Analysis (GSVA) method by R-packet (28). The limma package was used to screen the differences, and the relevant enrichment items with statistically significant differences were displayed by a heat map. Using the R-packet GSVA and single-sample Gene Set Enrichment Analysis (ssGSEA) methods combined with the 28 characteristic gene matrices of immune cells, we calculated the immune cell enrichment fraction of each sample.

### Weighted gene correlation network analysis

The aim (29) is to identify coexisting gene modules, explore the relationship between gene networks and phenotypes, and study the core genes in the network. Soft threshold was calculated by pick soft threshold function, and 5 were considered to be the optimal soft threshold. Scale-free networks were subsequently constructed based on soft thresholds, topology matrices were created, and hierarchical clustering was performed. Considering 50 as the minimum number of genes in the module, the identification gene module was dynamically cut and eigengenes were calculated. Based on eigengenes, the correlation between modules was constructed, and hierarchical clustering was carried out. The modules with a correlation above 0.7 were merged again, and lastly, 13 modules were obtained. The correlation between modules and clinical features was understood through Pearson’s correlation analysis.

### Construction of ceRNA network

CeRNA discloses the interaction mechanism among lncRNA, microRNA, and mRNA. The possible upstream miRNAs of TUBG1 and ABCC5 were searched through the mirTarbase database using multiMiR package (30). The StarBase database was used to download lncRNA–miRNA data (starBaseV3_hg19_CLIP-seq_LncRNA_all). Using pancancerNum >10 and clipExpNum>4 as criteria, lncRNAs are screened and intersected with those with significant differences in HCC data, and finally, four lncRNAs and 30 miRNAs are obtained. We completed the visual analysis using Cytoscape.

### PPI network building (STRING)

The STRING database (https://cn.string-db.org/) searches known and predicted protein interactions across 2,031 species, comprising 9.6 million proteins and 1.38 million PPIs. It includes results obtained from experimental data, PubMed Abstracts in Chinese, and the synthesis of data from other databases, as well as results predicted using bioinformatics methods. We used the STRING database to construct the PPI network of genes with common differences between the two diseases and set the parameter as correlation coefficient 0.7. The results from PPI are exported from the STRING database and visualized through Cytoscape. Furthermore, the CytoHubba plug-in was used to analyze Hub genes in the PPI network.

### Specimen collection

The study was approved by the Ethics Committee of the First Affiliated Hospital of Guangxi Medical University [NO.2022-KY-E-(115)]. NAFLD, HCC, and normal liver tissues were collected from tissue sections preserved in the Department of Pathology of our hospital. Informed consent was obtained.

### Cell model construction and cell culture

HepG2 and LO2 were cultured with Dulbecco's modified eagle medium (DMEM) (Multicell) containing 1% penicillin–streptomycin and 10% fetal bovine serum (FBS; Gibco) in a moist cell incubator at 37°C and 5% CO_2_.

High-fat LO2 cells and HepG2 cells (HF-LO2 group, HF-HepG2 group) were constructed by using sodium palmitate/sodium oleate (PA/OA) to simulate a high-free fatty acid environment. After the growth density of HepG2 and LO2 cells reached 70%–80% according to the manufacturer’s instructions, approximately 1 × 10^5^ cells were inoculated in 6-well plates and incubated for 24 h. The NAFLD model was established by a complete culture medium containing 500-μM sodium oleate and 250-μM sodium palmitate for 24 and 48 h. A complete culture medium supplemented with solvent was used as the control group (Control-LO2 group, Control-HepG2 group). The lipid accumulation level was detected by oil red O staining and triglyceride (TG). The TG assay kit (Jiancheng Bioengineering Institute, Nanjing, China) was utilized for extracting the contents of TG using the GPO-PAP method. According to the instruction, the cell homogenate and triglyceride assay reagents were added to the 96-well plate, mixed, and incubated at 37°C for 10 min, and the optical density (OD value) of each well was measured with the microplate reader (546 nm). The calculation formula was as follows: TG content = (sample OD value − blank OD value)/(calibration sample OD value − blank OD value) * Calibration sample concentration/sample protein concentration.

### RNA extraction and RT-QPCR

According to the manufacturer’s instructions, total RNA was isolated from cultured cells using a TRIzol reagent (TAKERA, Tokyo, Japan), and cDNA was obtained by reverse transcription using RT SuperMix for qPCR (Vazyme). The extracted cDNA was stored at -80°C. The target genes were amplified and detected using SYBR qPCR Master Mix (Vazyme, Nanjing, China) in the Applied Biosystems 7500 Fast real-time PCR system (ABI, Waltham, Massachusetts, USA). Primers for ABCC5 and Glyceraldehyde-3-Phosphate Dehydrogenase(GAPDH) are as follows: ABCC5-F (5’-ATC ATG GCT TGA GTG CTC TGA-3’) and ABCC5-R (5’-AGA CCA CAC GTC CAT TGA-3’); GAPDH-F (5’-AAT CAA GTG GGG CGA TGC TG-3’) and GAPDH-R (5’-GCA AAT GAG CCC CAG CCT TC-3’). The setting parameters were as follows: the denaturation temperature was 95°C for 15 min, the annealing temperature was 60°C for 1 min, and the extension was 40 cycles. The relative mRNA expression of ABCC5 was calculated by the 2^-ΔΔCT^ method (GAPDH as a housekeeping gene).

### Immunohistochemistry

Samples were prepared into paraffin sections, followed by dewaxing and hydration, and high-pressure and high-temperature antigen recovery in sodium citrate buffer (pH 6.0). Subsequently, it was incubated with endogenous peroxidase blockers and then incubated with a primary antibody for 12 h at 4°C. Sections were then rinsed with phosphate-buffered saline (PBS), followed by washing with an enzyme-coupled goat anti-rabbit/mouse IgG polymer for another 20 min and rinsed with PBS three times. Then, the sections were incubated with a DAB staining solution for 5 min. After washing with water, the paraffin sections were immersed in a hematoxylin- staining solution for 15 s and finally identified, rinsed, and returned to the laboratory for staining. The antibodies used included ABCC5 (Cat No. 19503-1-AP, 1:100; Proteintech, Wuhan, China) (31) and TUBG1 (Cat No. 15176-1-AP, 1:100; Proteintech, Wuhan, China) (32). Two pathologists observed the results. The number of positive cells was divided into five grades (0–4 score) according to the ratio (<5%, 5%–25%, 26%–50%, 51%–75%, 75%–100%), and the intensity of staining was divided into four grades (0–3 score). The group is obtained by multiplying the two fractions (from 0 to 12). A score of >4 was suggested to be positive.

### Statistical analysis

We performed all data calculations and statistical analysis using R programming (https://www.r-project.org/, version 4.0.2). The statistical significance of the normally distributed variables was calculated using an independent Student’s t-test, and the differences between the non-normally distributed variables were analyzed using the Mann–Whitney U test (i.e., Wilcoxon rank-sum test). All statistical p-values were bilateral, and P <0.05 was considered statistically significant.

## Results

### Weighted gene correlation network analysis consensus module identification

**Figure 1:**
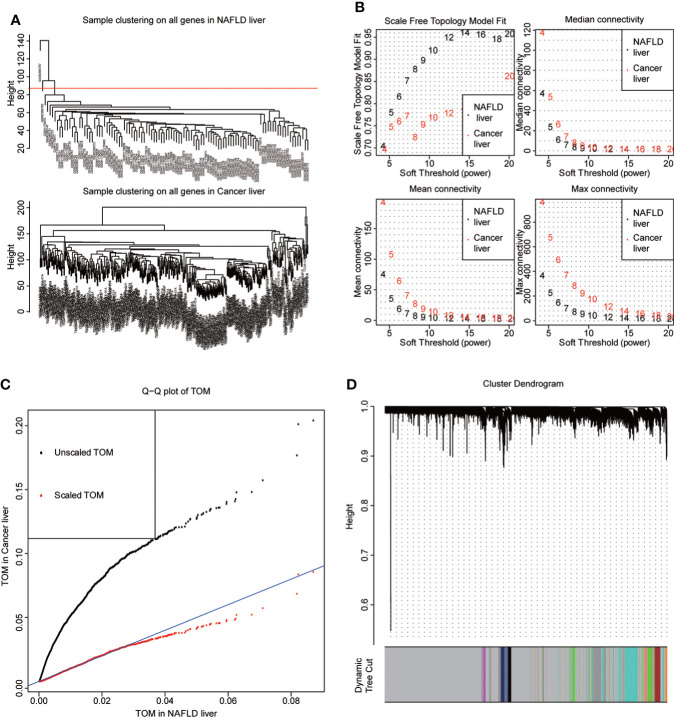
WGCNA. **(A)** The datasets of non-alcoholic fatty disease (NAFLD) and hepatocellular carcinoma (HCC) were based on the Euclidean distance clustering tree of samples. The red line of the dataset of NAFLD was the tangent line of outlier detection. Samples cut by this line are considered outliers. The tangents of the corresponding liver cancer samples are outside the tree, indicating that no samples from the HCC set are considered outliers. **(B)** Soft threshold power (x-axis) selection. NAFLD is approximately scale-free when the soft threshold is 7, while HCC is implemented at 20. With the increase of the soft threshold power, the comprehensive connectivity measure decreases sharply. Finally, we choose 7 as the soft threshold to construct the scale-free network. **(C)** Quantile–quantile plots of the dataset topological matrices (TOMs) for NAFLD and HCC. The black dots are TOMs before scaling, and the red dots are TOMs after scaling. After scaling, the two TOMs are comparable. **(D)** The gene tree was obtained by similarity clustering based on the overlap of consensus topology. As shown in the figure, there were 13 consensus gene modules in the two diseases, among which gray indicated unmatched genes.

**Figure 2:**
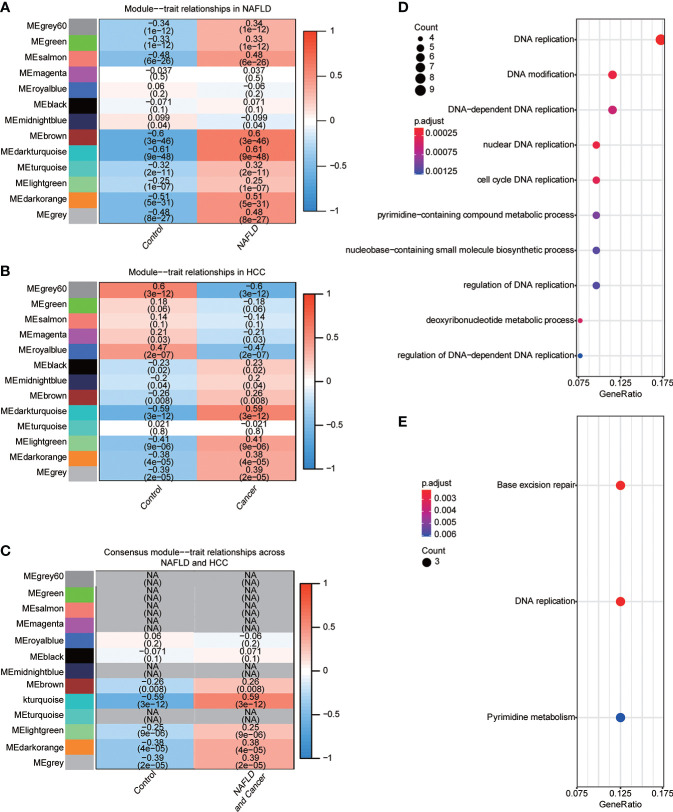
Identification of diagnostic-related modules. **(A)** Correlation analysis was used to find the correlation between 13 gene modules in NAFLD and whether they were sick or not. Red indicated positive correlation, blue indicated negative correlation, and P<0.05 was considered statistically significant. **(B)** Correlation analysis was used to find the correlation between 13 gene modules in HCC and whether they were sick or not. Red indicated positive correlation, blue indicated negative correlation, and P<0.05 was considered statistically significant. **(C)** The module dark turquoise is associated with the diagnosis of both diseases. **(D)** Gene Ontology (GO) function analysis of the dark turquoise module gene. **(E)** Kyoto Encyclopedia of Genes and Genomes (KEGG) function analysis of dark turquoise module gene.

NAFLD can ultimately cause HCC, but the mechanism by which hepatitis leads to HCC remains unclear. We assumed that the same gene modules expressed in both diseases might play a role in the copathogenesis of both diseases. NAFLD datasets GSE48452 and GSE89632 were downloaded from the GEO database and HCC dataset from the TCGA database. WGCNA analysis was used to identify gene modules that were expressed uniformly in both NAFLD and HCC. We removed large outliers with hierarchical clustering analysis (**Figure 1A**), scale-free networks and topological matrices were constructed by soft threshold (**Figure 1B**), and then topological overlapping matrices were scaled to make them comparable between two disease sets (**Figure 1C**). By extracting the minimum of parallel positions in two topological overlapping matrices [we now calculate the consensus topological overlap by taking the component-wise [“Parallel”)], the minimum of the topological matrices in individual sets acted as the consensus topological overlap matrix. Finally, we obtained 13 gene modules with similar expression in both diseases (**Figure 1D**). As clinical features, we explored modules related to the diagnosis of both diseases based on whether they were sick or not. The dark turquoise module had a high correlation and statistical significance with NAFLD and HCC among the 13 modules (**Figures 2A–C**). Then, we performed GO and KEGG function analysis on 58 genes in the dark turquoise module, and the results indicated that these genes were primarily involved in DNA-related enzyme activity, DNA shear, and the repair process (**Figures 2D, E**).

### Differences in gene

**Figure 3:**
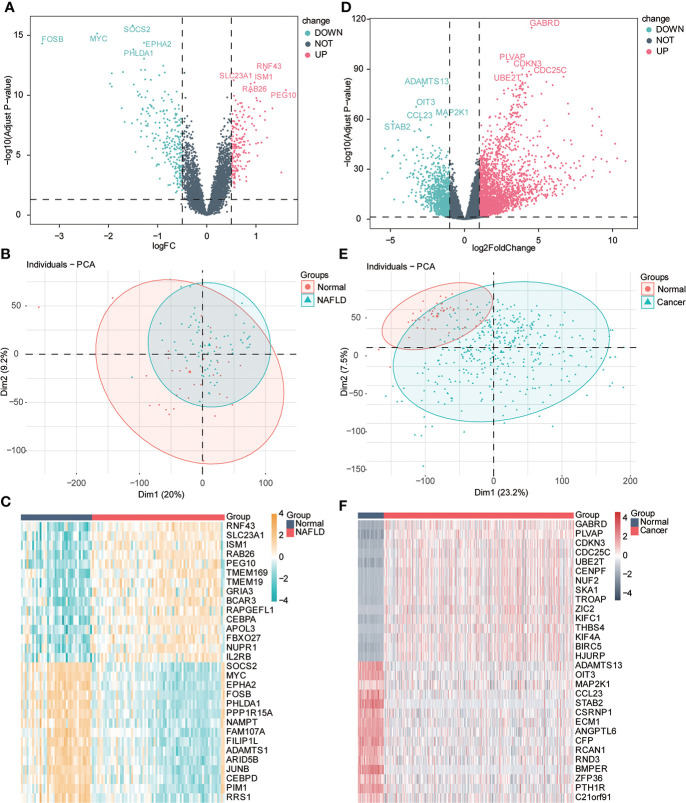
Variance analysis. **(A)** Volcanic diagram shows the differentially expressed genes (DEGs) of the NAFLD gene dataset, the horizontal axis is logFC value, the y-axis is -log10 (adjusted p-value), and the dotted line in the figure representslogFC> 0.5, adj-P < 0.05). Red represents upregulated genes, and blue represents downregulated genes. The first five gene labels are displayed in the adjusted p-value arrangement. **(B)** PCA principal component analysis of the NAFLD dataset: light green represents the samples of NAFLD, and red represents the samples of normal liver tissue (normal). **(C)** Heat map of differential genes. Red represents the sample of NAFLD, and dark blue represents the sample of normal liver tissue (normal). The top 20 genes are, respectively, visualized in adjusted p-value arrangement (yellow indicates high expression, and blue indicates low expression). **(D)** Volcanic diagram shows the DEGs of the HCC gene dataset; the horizontal axis is logFC value; the y-axis is -log10 (adjusted p-value), and the dotted line in the figure representslogFC> 1, adj. P < 0.05). Red represents upregulated genes, and blue represents downregulated genes. The first five gene labels are displayed in the adjusted p-value arrangement. **(E)** Principal component analysis (PCA) of HCC datasets: light green represents HCC tissue samples (cancer), and red represents normal liver tissue samples (normal). **(F)** Heat map of differential genes. Red represents HCC tissue samples (cancer), and dark blue represents normal liver tissue samples (normal). The top 20 genes are, respectively, visualized by adjusted p-value arrangement (red indicates high expression, and dark green indicates low expression).

We performed difference analysis on the two datasets to understand the correlation of transcriptional level changes in the two diseases. Using principal component analysis (PCA), we found some differences in expression profiles between the NAFLD group and the normal group. We used the limma package for difference analysis to obtain DEGs in the two data groups, including 181 upregulated genes and 232 downregulated genes, which were visualized by the volcanic diagram and heat maps (**Figures 3A–C**). Significant differences were found between HCC and the control group gene expression profiles. We used the Deseq2 package to analyze the differences of genes in different groups, comprising 2,490 upregulated genes and 1,077 downregulated genes, which were also used and visualized by the volcano map and heat map (**Figures 3D–F**).

**Figure 4:**
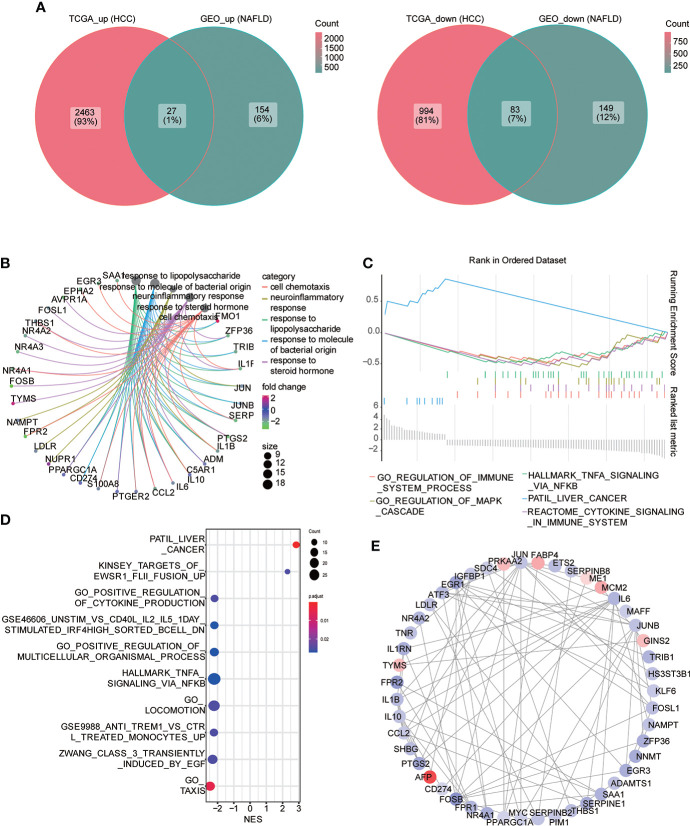
Common differential genes. **(A)** Venn diagram shows the coupregulated and codownregulated genes in the two diseases. **(B)** Network diagram showing the GO function analysis of common differential genes. **(C)** Gene set enrichment analysis (GSEA) of common differential genes: the logFC value was used to arrange genes, and the GSEA algorithm was used to calculate the first two enrichment items and the last two enrichment items in descending order. **(D)** GSEA analysis: the top 10 enrichment pathway were visualized in the bubble diagram, arranged with the descending order of the absolute value of enrichment fraction (NES). **(E)** PPI network construction, from red to blue; continuous color change represents the logFC value from high to low.

Subsequently, we searched for both upregulated and downregulated genes in the two diseases and demonstrated them through the Venn diagram. The results showed that 27 genes were upregulated in the two diseases compared with the control group, while 83 genes were downregulated in the two diseases compared with the control group (**Figure 4A**). We performed the GO function analysis of genes to further understand the biological function of these genes with same trend. The results indicated that these genes were mainly involved in cell chemotaxis, neuroresponse, response to lipopolysaccharide, response to the molecule of bacterial origin, response to steroid hormone; a variety of lipid-related functional items are involved, and then we used the mesh diagram to visualize the main items (**Figure 4B**; **Supplement Table 1**). Then, we used GSEA enrichment analysis to enrich the pathway-related dataset in MSigDb based on the above gene list. The results indicated that it was associated with multiple immune, HCC, and cancer-related pathways (**Figures 4C, D**; **Supplement Table 2**). Consequently, we constructed the PPI network through STRING (correlation coefficient 0.7) and visualized it using Cytoscape (**Figure 4E**). The top three genes interacting with other proteins were EGR1, FOSB, and CCL2.

### To construct a diagnostic model of non-alcoholic fatty liver disease

**Figure 5:**
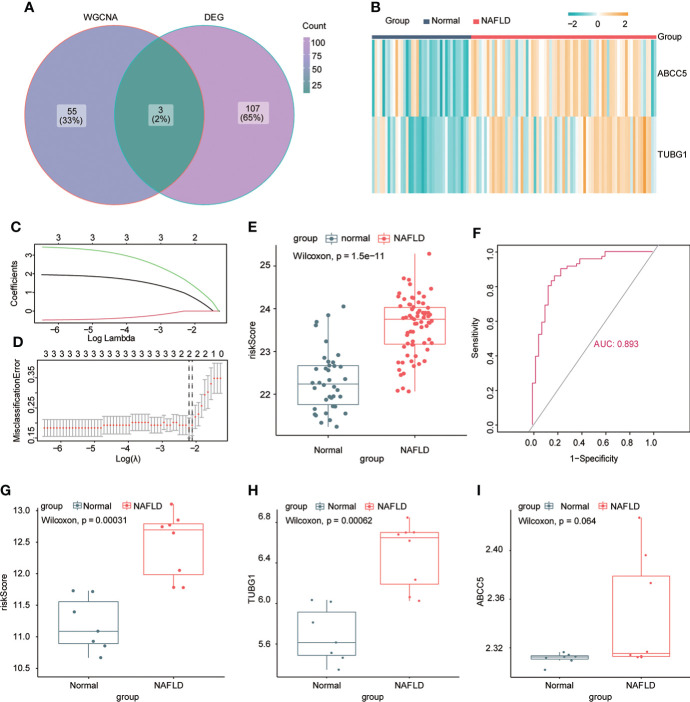
Establishment of a diagnostic model of NAFLD. **(A)** Venn diagram shows the coexisting genes (ABCC5, DHODH, TUBG1) of the diagnosis-related gene module darkturquoise in WGCNA and common differential genes. **(B)** Visualizing the expression profile of ABCC5 and TUBG1 in NAFLD by a heat map. **(C)** LASSO model tuning parameters were selected through 10-fold cross-validation. **(D)** LASSO coefficient spectra of diagnosis-relevant gene sets with dotted lines representing values selected by 10 cross-validations. A diagnostic model containing two genes (ABCC5 and TUBG1) was obtained. **(E)** Box plot presenting the difference in the risk score between the NAFLD group and the normal group. **(F)** ROC curve confirms that the diagnostic model has high diagnostic efficiency (AUC = 0.893). **(G)** Based on the new dataset (GSE37031), the risk score was calculated according to the model; the risk value in the NAFLD group was significantly higher than that in the normal group. **(H)** The expression level of TUBG1 in the GSE37031 dataset visualized by box plot in the NAFLD group and normal liver tissue sample group (P<0.05 is considered statistically significant). **(I)** Box plot visualization of ABCC5 expression in the GSE37031 dataset between the NAFLD group and the normal group.

The consensus module genes related to the diagnosis of the two diseases obtained by WGCNA were intersected with the different genes (DEG) with the same change trend in the two diseases to find efficient diagnostic and therapeutic markers (**Figure 5A**), suggesting three coexisting genes (ABCC5, DHODH, and TUBG1). After univariate and multivariate logistic regression analyses, ABCC5 and TUBG1 genes were found to be independent risk factors for the diagnosis of NAFLD and HCC (OR>1; P <0.05), while DHODH was an independent protective factor in HCC (OR<1; P < 0, 05) (**Tables 1**, **Table 2**). These findings propose that ABCC5 and TUBG1 may have certain roles in the occurrence and development of the two diseases. We visualized the expression of the two genes by a heat map in the group of NAFLD (**Figure 5B**). The above three genes were reverified using minimal absolute contraction and selection operator LASSO regression. The results were in line with multivariate logistic regression. Two genes, ABCC5 and TUBG1, were involved in constructing a diagnostic model of NAFLD (**Figures 5C, D**). Its correlation coefficient was used to calculate the risk values of all samples, and a box plot was used to visualize the difference of risk values between the NAFLD group and the normal group, indicating that the risk values of NAFLD were significantly higher (**Figure 5E**). According to the ROC curve results, the diagnostic model constructed by ABCC5 and TUBG1 genes had very good diagnostic efficiency (AUC = 0.893) (**Figure 5F**). As further validation of our results, we downloaded the NAFLD dataset GSE37031 through the GEO database. The results showed that the risk value of the NAFLD group was significantly higher, and the TUBG1 expression level was also higher in the NAFLD group (P<0.01) (**Figures 5G, H**). While the box plot indicated that the ABCC5 expression level was high in the NAFLD group (P = 0.064), the p-value was insignificant as well as the AUC value of the ROC curve was 1, which may result from the small sample size. (**Figure 5I**, **Supplement Figure 1**). Results from these studies suggest that the high expression of ABCC5 and TUBG1 is an independent risk factor for NAFLD and can be used as a diagnostic indicator.

**Table 1:** Univariate and multivariate logistic regression (non-alcoholic fatty liver disease).

	Univariate analysis		Multivariate analysis	
	OR (95% CI)	P value	OR (95% CI)	P-value
ABCC5	18.133 (5.445–60.391)	<0.001	7.188 (1.900–27.196)	0.004
DHODH	0.195 (0.090–0.426)	<0.001	NA	
TUBG1	63.821 (13.06–311.663)	<0.001	32.515 (5.976–176.904)	0

**Table 2:** Univariate and multivariate logistic regression (The Cancer Genome Atlas).

	Univariate analysis		Multivariate analysis	
	OR (95% CI)	P-value	OR (95% CI)	P-value
ABCC5	40.238 (15.785–102.571)	<0.001	5.514 (1.884–16.135)	0.002
DHODH	0.245 (0.167–0.358)	<0.001	0.429 (0.247– 0.744)	0.003
TUBG1	44.468 (16.669–118.632)	<0.001	13.254 (4.369–40.214)	0

### ABCC5 and TUBG1 are independent risk factors for diagnosis of hepatocellular carcinoma

By performing univariate and multivariate logistic regression analysis on three coexisting genes (ABCC5, DHODH, and TUBG1) in TCGA HCC dataset, it was found that ABCC5 and TUBG1 were independent risk factors for HCC (OR>1; P <0.05), whereas DHODH was an independent protective factor in HCC (OR<1; < 0, 05) (**Table 2**). To further confirm our conclusions, we used HCC dataset (LIRI-JP) from the ICGC database for validation and found ABCC5 and TUBG1 genes were independent risk factors for HCC (OR>1; P <0,05), while DHODH was not (**Table 3**). These outcomes showed that ABCC5 and TUBG1 were independent risk factors for NAFLD and HCC, suggesting that the two genes possessed potential diagnostic value in both diseases.

**Table 3:** Univariate and multivariate logistic regression (ICGC).

	Univariate analysis		Multivariate analysis	
	OR (95% CI)	P-value	OR (95% CI)	P value
ABCC5	10.444 (6.676–16.339)	<0.001	2.109 (1.197- 3.715)	0.01
DHODH	0.353 (0.281–0.445)	<0.001	NA	
TUBG1	50.090 (24.340–103.081)	<0.001	26.418 (12.231–57.063)	0

### Construction of prognostic model in hepatocellular carcinoma

**Figure 6:**
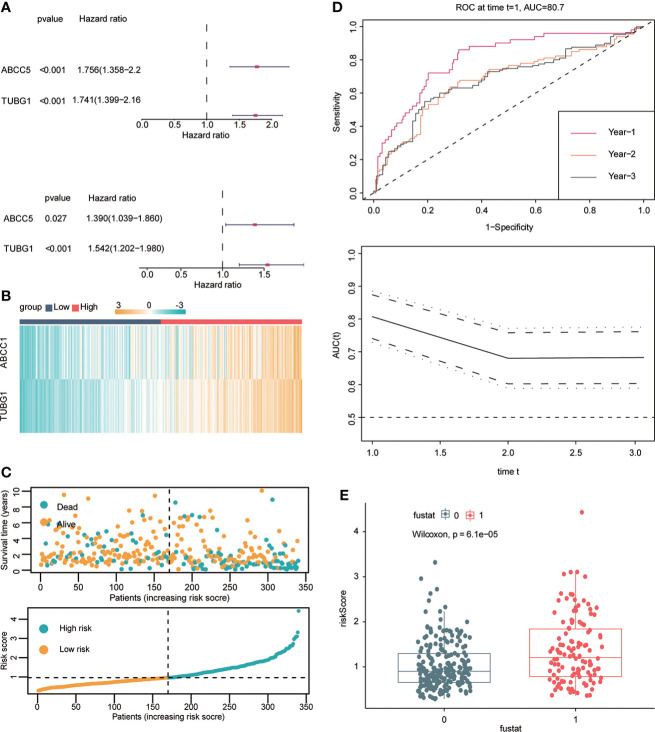
Construction of hepatocellular carcinoma prognostic model. **(A)** Univariate Cox analysis suggested that ABCC5 and TUBG1 were correlated with the prognosis of HCC. Multivariate Cox analysis suggested that ABCC5 and TUBG1 were independent risk factors (red represents HR>1; dark blue represents HR<1, P<0.05 is statistically significant). **(B)** Both ABCC5 and TUBG were upregulated in the high-risk group (red represents high-risk group; dark blue represents low-risk group). **(C)** The risk value was calculated by multivariate Cox regression. With the increase of the risk value, the survival time decreased, and the number of patients who died increased. **(D)** With risk score as the observed value, the time ROC curve confirmed that it had good diagnostic value at 1-year, 2-year, and 3-year survival nodes (the AUC of a 1-year ROC curve was 0.8, the 2-year ROC curve was 0.68, and the 3-year ROC curve was 0.68). **(E)** The box plot shows that the death group has higher risk scores (0 represents alive group; 1 represents dead group).

**Figure 7:**
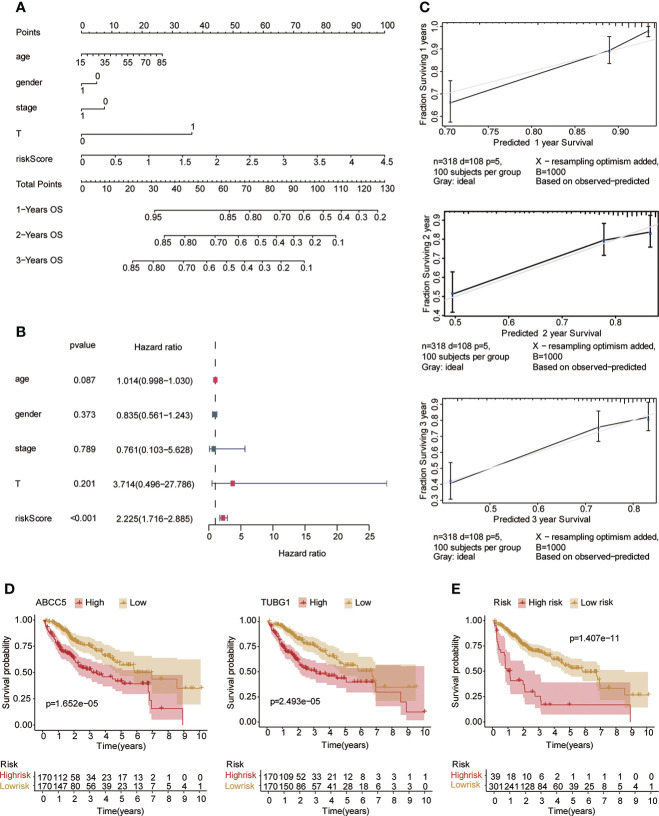
Risk model testing. **(A)** Nomogram recommends that risk score would contribute the most than other clinical characteristics (age, gender, stage, and the depth of invasion). **(B)** Multivariate Cox regression analysis of the risk score combined with clinical characteristics (age, gender, stage, and the depth of invasion) suggested that the risk score was still an independent risk factor. **(C)** The results of 1-year, 2-year, and 3-year calibration curves show that the prediction accuracy of risk models is high. **(D)** The survival curve suggested that patients in both ABCC5 and TUBG1 high-expression groups had a poor prognosis (red represents a high-expression group; yellow represents low-expression group). **(E)** The survival curve suggested that patients in the high-risk group had a poor prognosis (red represents a high-risk group; yellow represents low-risk group, P<0.05 is statistically significant).

**Figure 8:**
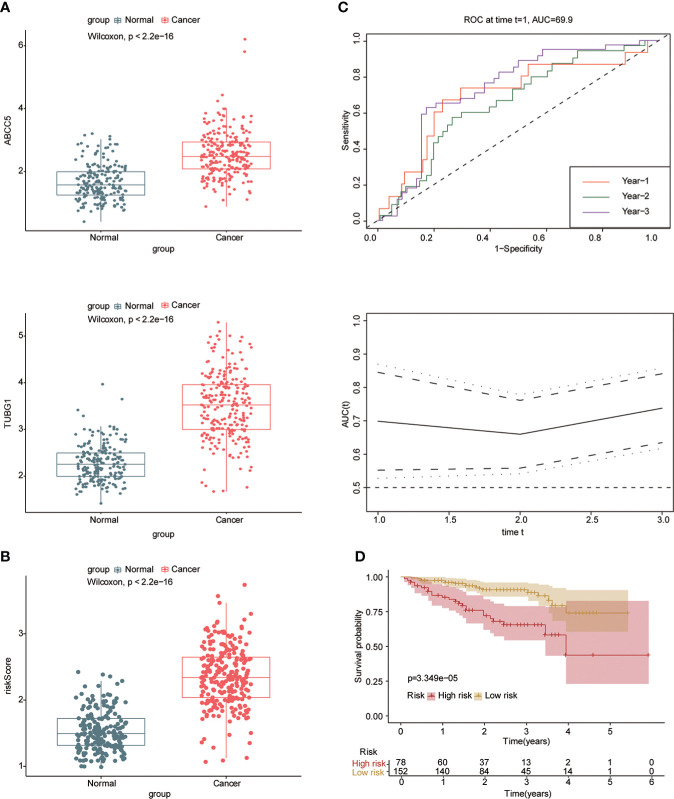
Risk model was tested with the ICGC-HCC dataset. **(A)** The box plot shows that the expression of ABCC5 and TUBG in the ICGC-HCC dataset weas significantly higher in the HCC group. **(B)** The box plot shows that in the ICGC-HCC dataset, the risk score in the HCC group was significantly higher than that in the normal group. **(C)** With the risk score as the observed value, the timeROC curve confirmed that our prognostic model still had a high diagnostic value (the AUC of the 1-year ROC curve was 0.7, the 2-year ROC curve was 0.66, and the 3-year ROC curve was 0.74). **(D)** The survival curve suggested that patients in the high-risk group had a poor prognosis (red represents high-risk group; yellow represents low-risk group, P<0.05 is statistically significant).

Univariate and multivariate Cox regression analyses were performed to explore whether three coexisting genes (ABCC5, DHODH, and TUBG1) were associated with the prognosis of HCC, and the results showed that ABCC5 and TUBG1 were independent risk factors for the prognosis of HCC (HR>1, P<0.05) (**Figure 6A**). Subsequently, we calculated the risk value of each sample based on the correlation coefficient. There were more deaths, shorter survival time, and higher expression levels of ABCC5 and TUBG1 in the high-risk group (**Figures 6B, C**). Afterward, we verified our prognostic model using ROC curves with survival time nodes of 1, 2, and 3 years. The results presented the AUC value of 1-year, 2-year, and 3-year ROC curves to be 0.80, 0.68, and 0.68, respectively (**Figure 6D**). As shown in **Figure 6E**, the risk value was higher in the death group (P<0.05). Using the nomogram, we found that when combined with age, gender, stage, and the depth of invasion, the risk score significantly contributed to survival risk (**Figure 7A**). We performed a multivariate Cox analysis in combination with clinical features to determine the influence of clinicopathological features on riskScore. The findings indicated that the predictive model’s riskScore remained an independent risk factor in combination with other clinical features (HR = 2.225 95%, CI [1.716–2.885]; P < 0.001) (**Figure 7B**). Next, we tested the predictive accuracy of the model using the calibration curve, and the results showed that the prediction accuracy of 1-year, 2-year and 3-year survival rates was very high (**Figure 7C**). The survival curve showed that both ABCC5 and TUBG1 were significantly correlated with poor prognosis in HCC and the survival rate of patients in the high-risk group was significantly lower than that in the low-risk group (**Figures 7D, E**). So as to further verify our prognostic model, HCC-related data (LIRI-JP) from the ICGC database were used for confirmation. The ABCC5 and TUBG1 expressions were significantly increased in the HCC group. In the meantime, based on the model, it was determined that the HCC group had a significantly higher risk score than the normal group (**Figures 8A, B**). Then, we verified our prognostic model by using ROC curves with survival time nodes of 1, 2, and 3 years. The results disclosed that the AUC of 1-year, 2-year and 3-year ROC curves was 0.70, 0.66, and 0.74, respectively (**Figure 8C**). Meanwhile, the survival curve showed that high risk score was significantly correlated with poorPrognosis in LIRI-JP (**Figure 8D**).

### Changes of immune microenvironment and construction of ceRNA network

**Figure 9:**
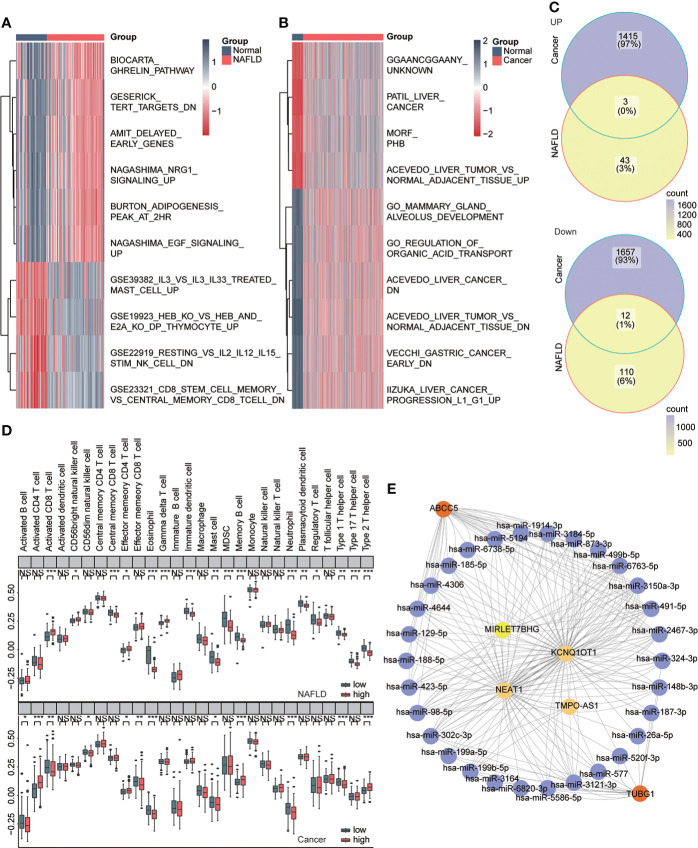
Immune infiltration and ceRNA. **(A)** Heat map showing pathways that differ significantly between the normal and NAFLD groups (red for NAFLD group and dark blue for normal group). **(B)** Heat map shows pathways with significant differences between the normal group and the HCC group (red represents the cancer group; dark blue represents the normal group). **(C)** Venn diagram shows coupregulated and codownregulated pathways in both diseases. **(D)** The upper box plot shows the immune infiltration of 28 cells in the high- and low-risk group of NAFLD, and the lower box diagram shows the immune infiltration of 28 cells in in the high- and low-risk group of HCC. **(E)** ABCC5 and TUBG1-related ceRNA networks were constructed. Red represents mRNA, purple represents microRNA, and yellow represents lncRNA.

We used the GSVA algorithm to analyze pathway-related datasets in MSigDb in the two diseases and the limma package for difference analysis. LogFC = 0.5, ADj. P<0.05 were used as thresholds to screen related pathways with significant differences between the disease and normal groups. The results showed 46 significantly upregulated and 122 significantly downregulated pathways in NAFLD and 1,418 significantly upregulated and 1,669 significantly downregulated pathways in HCC. We visualized the top 10 pathways with significant changes using heat maps (**Figures 9A, B**) and then obtained both upregulated and downregulated pathways in the two diseases (**Figure 9C**, **Table 4**). The outcomes showed that NAFLD and HCC had different fat metabolism, immunity, tumor, and other related pathways as compared to the normal group. These pathways may disclose the principal mechanisms by which NAFLD progresses to HCC. We conducted enrichment analysis of 28 immune cell components in the two datasets ssGSEA to understand the immune infiltration of the two diseases and found that the immune microenvironment of the two diseases had significantly changed. Furthermore, effector memory CD4 T cells, eosinophils, mast cells, neutrophils, and type 1 T helper cells showed significant differences and the same trend in the high- and low-risk groups of the two diseases (**Figure 9D**). CeRNA discloses the interaction mechanism among lncRNA, microRNA, and mRNA. The upstream miRNAs and lncRNAs of ABCC5 and TUBG1 were predicted through the ceRNA network, and visualization analysis was executed using Cytoscape (**Figure 9E**). According to the ceRNA mechanism, lncRNAs can upregulate the expression of their target mRNA. Hence, we used the upregulated lncRNAs expressed in HCC data based on TCGA and intersected with the predicted lncRNAs to find four lncRNAs with high confidence and consistent expression.

**Table 4:** GSVA: both upregulated and downregulated pathways occur in both diseases.

	GSVA
UP	GO_LONG_CHAIN_FATTY_ACYL_COA_BINDING
UP	BIOCARTA_TCAPOPTOSIS_PATHWAY
UP	GO_WATER_SOLUBLE_VITAMIN_BIOSYNTHETIC_PROCESS
DOWN	BIOCARTA_GHRELIN_PATHWAY
DOWN	TIAN_TNF_SIGNALING_NOT_VIA_NFKB
DOWN	AMIT_SERUM_RESPONSE_40_MCF10A
DOWN	PHONG_TNF_TARGETS_UP
DOWN	GRAHAM_CML_QUIESCENT_VS_CML_DIVIDING_UP
DOWN	AMIT_EGF_RESPONSE_20_HELA
DOWN	GO_POSITIVE_REGULATION_OF_CARDIOBLAST_DIFFERENTIATION
DOWN	GO_NEGATIVE_REGULATION_OF_PLASMINOGEN_ACTIVATION
DOWN	FUNG_IL2_TARGETS_WITH_STAT5_BINDING_SITES_T1
DOWN	GO_POSITIVE_REGULATION_OF_SARCOMERE_ORGANIZATION
DOWN	REACTOME_RUNX1_REGULATES_TRANSCRIPTION_OF_GENES_INVOLVED_IN_INTERLEUKIN_SIGNALING
DOWN	GO_SNORNA_LOCALIZATION

### Expression of ABCC5 and TUBG1 at tissue and cell levels

**Figure 10:**
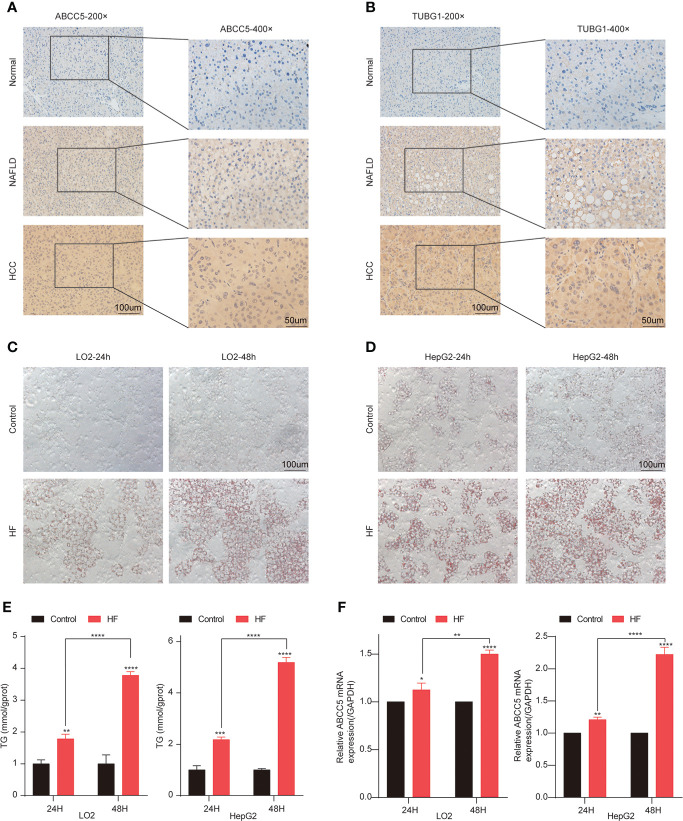
Lipidization increased the expression of ABCC5 in LO2 and HepG2. **(A)** The expression of ABCC5 in human normal liver tissue, NAFLD tissues and HCC tissues (magnification ×200, scale bars 100 μm; magnification ×400, scale bars 50 μm). **(B)** The expression of TUBG1 in human normal liver tissue, NAFLD tissues, and HCC tissues (magnification ×200, scale bars 100 μm; magnification ×400; scale bars 50 μm). **(C)** The Oil Red O staining of LO2 cells treated with or without sodium palmitate/sodium oleate (PA/OA) for 24 h (magnification ×200, scale bars 100 μm). **(D)** The Oil Red O staining of HepG2 cells treated with or without PA/OA for 48 h (magnification ×200, scale bars 100 μm). **(E)** TG content in HepG2 cells and LO2 cells treated with PA/OA for 24 and 48 h. **(F)** The mRNA expression of ABCC5 examined by RT-qPCR. ([HF] represents high-fat group) (P < 0.05 *, P <0.01 **, P <0.001 ***, P <0.0001 ****).

Subsequently, the expression of ABCC5 and TUBG1 in normal liver tissue, NAFLD tissue, and HCC tissue was detected by immunohistochemistry. The results showed that compared with normal liver tissue, the protein expression levels of ABCC5 and TUBG1 in NAFLD and HCC tissues were significantly increased (P <0.05) (**Figures 10A, B**).

In order to further explore the expression of ABCC5 and TUBG1 in NAFLD and HCC. We simulated a high-fatty-acid environment by mixing PA and OA (33). HF-LO2 group and HF-HepG2 group models were constructed (34–36). Oil red O staining results showed that OA significantly increased lipid accumulation in HepG2 cells and LO2 cells compared with the control group (**Figures 10C, D**). The results of the quantitative analysis of triglyceride were consistent with the above results, and the triglyceride content of cells in HF-LO2 and HF-HepG2 groups was significantly higher than that in the corresponding control group (**Figure 10E**). The above results indicated that the adipose cell model was successfully constructed.

The transcriptional levels of ABCC5 and TUBG1 in HepG2 cells and LO2 cells were detected by RT-QPCR. The results showed that ABCC5 expression was significantly increased in HepG2 cells compared with LO2 cells (P <0.05). Similarly, RT-QPCR results showed that ABCC5 expression was significantly increased in both the HF-LO2 group and the HF-HepG2 group after aliphication, and the increased level increased with the extension of aliphication time (P <0.05) (**Figure 10F**). However, the transcription level of TUBG1 was not significantly changed in HepG2 and LO2 cell lines and before and after adipose treatment.

## Discussion

The annual global increase in the HCC incidence is inseparable from NAFLD, an independent risk factor. In the wake of increasing living standards, the incidence of NAFLD is increasing at an alarming speed. The prevalence of NAFLD is increasing at an alarming rate and has been considered an inducible cause of HCC (37), and non-alcoholic steatohepatitis (NASH), as a manifestation of NAFLD, can progress to cirrhosis or even HCC (38). Elucidating the mechanism of NAFLD to HCC will enable us to have the opportunity to take measures at the early stage, so as to achieve early prevention and reduce the incidence of HCC, which will be of great significance.

Currently, some excellent studies have tried to explore the underlying mechanism of the progression of NAFLD to HCC. Dabin Liu (39) et al. found that SQLE (squalene epoxidase) may play an important role in the progression of NAFLD to HCC. The authors found that SQLE was abnormally upregulated in both NAFLD and HCC. *In vitro* and *in vivo* experiments have confirmed that abnormal expression of SQLE leads to a significant increase in intracellular cholesterol esters and induces oxidative stress. Increased intracellular cholesterol esters are associated with the growth and invasion of a variety of tumors, while oxidative stress can activate multiple downstream tumor-related pathways, such as the PI3K/AKT/mTOR signal pathway. Meanwhile, the authors also demonstrated that terbinafine may exert its antitumor effect by targeting SQLE. However, the pathogenesis of HCC induced by NAFLD still stays uncleared. Therefore, we explored the potential mechanism of NAFLD to HCC and found two key genes.

In this study, 26 upregulated and 87 downregulated genes were obtained by limma package analysis using the NAFLD dataset (GSE48452, GSE89632, GSE37031) and HCC dataset TCGA-LIHC (n = 445 cases). By using the limma package to analyze the differences in the data of the two diseases, 26 coupregulated genes and 87 codownregulated genes were obtained. Then, we used 13 consensus modules screened by WGCNA from the two diseases, among which the darkturquoise module was highly correlated with the diagnosis of both diseases. The intersection of the dark turquoise module and common downregulated genes was obtained, and the coexpressed genes ABCC5, DHODH, and TUBG1, which were expressed uniformly in both diseases and were related to diagnosis, were finally obtained. After the establishment of diagnostic and prognostic models and the verification process of prediction ability, it was concluded that the expression patterns of ABCC5 and TUBG1 in NAFLD and HCC were consistent, which were common diagnostic markers and highly correlated with the prognosis of HCC. Both may be closely related to NAFLD-induced HCC and may become new biomarkers for the biological diagnosis and prognosis of HCC. Current studies indicate that ABCC5 plays an oncogene role in HCC. Borel et al. (40) studied the expression of 15 ABC transporters related to multidrug resistance in 19 samples of HCC patients (16 untreated and 3 treated with chemotherapy); 12 ABC transporters were found to be upregulated in HCC as compared to healthy livers, and one of them is ABCC5. Chen has found that (41)ABCC5 levels are significantly increased in HCC, and it is considered to be an independent risk factor for HCC progression and prognosis mainly because of immune cell infiltration. Due to its drug resistance characteristics, Huang et al. (42) proved that the regulation of the PI3K/AKT/NRF2 pathway activates and upregulates ABCC5 to promote the resistance of sorafenib (a first-line molecular targeted drug for advanced HCC) acquired in human HCC cells. This result suggests that ABCC5 is an important regulator and a promising therapeutic target of sorafenib resistance acquired in human HCC cells. ABCC5 has also been found to be able to regulate and reduce GPx4 consumption in sorafenib-resistant HCC cells to inhibit lipid peroxidation. Additionally, ABC transporters mainly mediate the transmembrane transport of cyclic nucleotides or nucleotide analogs, able to transport organic anionic compounds, including glucoside acids, and the sulfate conjugates of steroids (43). Further studies (44) have found that it actively transports bile acids made by cholesterol in liver cells. Lyu (45), Liu (46), and Akbulut (47) et al. further demonstrated that ABCA1 could mediate the transport of cholesterol and phospholipids from cells to high-density lipoprotein apolipoprotein, thereby affecting the transport of liver cholesterol and inducing fatty liver disease. Hardwick et al. (16) suggested that six ABC transporters, including ABCC5, ABCB1, and ABCG2, showed an increasing trend in the progression of NAFLD. In support of this theory, Cyranka et al. (48) demonstrated that ABCC5 gene knockout mice had reduced white and brown adipose tissue, and the most prominent metabolic phenotype was the reduction of total adipose mass, suggesting a new concept that ABCC5 protein expression plays a key role in mammalian energy metabolism.

The results of our bioinformatics analysis are consistent with the above studies. ABCC5 is a diagnostic marker in both diseases and is associated with the prognosis of HCC. In addition, it was found for the first time *in vitro* that the expression level of ABCC5 was significantly increased in hepatocellular carcinoma after adipose transformation, and the increased level was positively correlated with the degree of adipose transformation.

TUBG1 is a member of the human tubulin family and is involved in the development of various tumors. In the study of Hubert (49) and Hsu (50) et al. it is found the breast Cancer 1 protein (BRCA1), an inhibitor of breast and ovarian tumorigenesis, maintained a high fidelity of cell division after centrosome binding to TUBG1 produced a ubiquitination reaction. Furthermore, its association with tumor suppressor P53 and centrosome was explained by Morris (51) and Kanai (52) et al. It was also revealed by Hořejší et al. that high expression of TUBG1 antagonized its inhibitory effect on DNA damage (53). In a hospital-based case–control study (54), variations in TUBG1 were also associated with breast cancer risk. Blanco et al. (55) further explored that TUBG1 interacts with associated proteins in sporadic breast tumors to regulate the mammary epithelial polarization and affect patient survival. However, the relationship between TUBG1 and the progression of HCC and NAFLD patients is still unclear, and few studies have been conducted in this context. Through bioinformatics multiple analysis, we believe that this gene may be a common carcinogenic factor in the pathogenesis of NAFLD and HCC. Our immunohistochemical results were consistent with the results of the bioassay, and the TUBG1 expression level was significantly increased in both NAFLD and HCC tissues. However, there was no significant change in the transcription level after fatty treatment. We speculated that the increase of the TUBG1 protein level may not be caused by its own increase but rather due to changes in the microenvironment of liver cells under a high-lipid environment. We believe that TUBG1 is a very promising research site.

Although we verified the above results by using a variety of methods, there are still limitations. The regulatory mechanism of ABCC5 and TUBG1 in the development of HCC in the context of NAFLD is not detailed yet, and whether the differences of the last two genes in different stages of NAFLD-HCC progression also needs further study and confirmation.

Our results showed that ABCC5 and TUBG1 were increased in both NAFLD and HCC and were independent risk factors. The diagnostic model of NAFLD and the prognostic model of HCC jointly constructed by ABCC5 and TUBG1 had high predictive ability. Both may be closely related to NAfLD-HCC. We found for the first time that a high-fat environment promotes the expression of ABCC5 in HCC, further elucidating the carcinogenic mechanism of ABCC5 in HCC and its possible important role in the pathogenesis of NAFLD-HCC.

Furthermore, we also explored the immune cells with the same changing trend of the immune microenvironment in NAFLD and HCC, as well as the common up–downregulation pathway, providing new clues for exploring the pathogenesis of NAFLD-HCC. Simultaneously, referring to the studies that established that lncRNA- D16366 (56)and circ_0067934 (57) could be used as novel diagnostic and prognostic markers of HCC, the upstream miRNAs and lncRNAs of ABCC5 and TUBG1 were predicted through the ceRNA network. In conclusion, ABCC5 and TUBG1 are likely to become copathogenic factors of NAFLD and HCC, opening a new window of opportunity for individualized and precise diagnosis, prevention, and treatment of NAFLD.

## Data availability statement

The datasets presented in this study can be found in online repositories. The names of the repository/repositories and accession number(s) can be found in the article/**Supplementary Material**.

## Author contributions

TC, XY, and SZ contributed to the study conception and design. Material preparation, data collection, and analysis were performed by TC, SZ, DZ, PL, XM, and RT. The first draft of the manuscript was written by TC, and all authors commented on previous versions of the manuscript. All authors read and approved the final manuscript. XY contributed to check this manuscript. All authors contributed to the article and approved the submitted version.

## Funding

This work was supported in part by the Science and Technology Plan Project of Qingxiu District, Nanning City [grant no. 2019024], ‘Medical Excellence Award’ Funded by the Creative Research Development Grant from the First Affiliated Hospital of Guangxi Medical University [grant no. 2017025], The basic ability improvement project for young and middle-aged teachers in Guangxi universities [grant no. 2017KY0100], and Medical and Health Appropriate Technology Development, Promotion and Application Project of Guangxi [grant no. 2016018].

## Conflict of interest

The authors declare that the research was conducted in the absence of any commercial or financial relationships that could be construed as a potential conflict of interest.

## Publisher’s note

All claims expressed in this article are solely those of the authors and do not necessarily represent those of their affiliated organizations, or those of the publisher, the editors and the reviewers. Any product that may be evaluated in this article, or claim that may be made by its manufacturer, is not guaranteed or endorsed by the publisher.

## Supplementary material

Supplementary Figure 1 The ROC curve of risk score based on GSE37031

## References

[1] Uzunlulu M Telci Caklili O Oguz A. Association between metabolic syndrome and cancer. Ann Nutr Metab (2016) 68:173–9. doi: 10.1159/000443743 26895247

[2] Yu J Shen J Sun TT Zhang X Wong N. Obesity, insulin resistance, NASH and hepatocellular carcinoma. Semin Cancer Biol (2013) 23:483–91. doi: 10.1016/j.semcancer.2013.07.003 23876851

[3] Baffy G Brunt EM Caldwell SH. Hepatocellular carcinoma in non-alcoholic fatty liver disease: an emerging menace. J Hepatol (2012) 56:1384–91. doi: 10.1016/j.jhep.2011.10.027 22326465

[4] Sanyal A Poklepovic A Moyneur E Barghout V. Population-based risk factors and resource utilization for HCC: US perspective. Curr Med Res Opin (2010) 26:2183–91. doi: 10.1185/03007995.2010.506375 20666689

[5] Younossi ZM Koenig AB Abdelatif D Fazel Y Henry L Wymer M. Global epidemiology of nonalcoholic fatty liver disease-meta-analytic assessment of prevalence, incidence, and outcomes. Hepatology (2016) 64:73–84. doi: 10.1002/hep.28431 26707365

[6] Mittal S El-Serag HB Sada YH Kanwal F Duan Z Temple S. Hepatocellular carcinoma in the absence of cirrhosis in united states veterans is associated with nonalcoholic fatty liver disease. Clin Gastroenterol Hepatol (2016) 14:124–31.e121. doi: 10.1016/j.cgh.2015.07.019 26196445 PMC4690789

[7] Heimbach JK Kulik LM Finn RS Sirlin CB Abecassis MM Roberts LR. AASLD guidelines for the treatment of hepatocellular carcinoma. Hepatology (2018) 67:358–80. doi: 10.1002/hep.29086 28130846

[8] Fujiwara N Friedman SL Goossens N Hoshida Y. Risk factors and prevention of hepatocellular carcinoma in the era of precision medicine. J Hepatol (2018) 68:526–49. doi: 10.1016/j.jhep.2017.09.016 PMC5818315 28989095

[9] Donati B Pietrelli A Pingitore P Dongiovanni P Caddeo A Walker L. Telomerase reverse transcriptase germline mutations and hepatocellular carcinoma in patients with nonalcoholic fatty liver disease. Cancer Med (2017) 6:1930–40. doi: 10.1002/cam4.1078 PMC5548883 28677271

[10] Chen J Chen J Huang J Li Z Gong Y Zou B. HIF-2α upregulation mediated by hypoxia promotes NAFLD-HCC progression by activating lipid synthesis *via* the PI3K-AKT-mTOR pathway. Aging (Albany N Y) (2019) 11:10839–60. doi: 10.18632/aging.102488 PMC6932893 31796646

[11] Sobolewski C Abegg D Berthou F Dolicka D Calo N Sempoux C. S100A11/ANXA2 belongs to a tumour suppressor/oncogene network deregulated early with steatosis and involved in inflammation and hepatocellular carcinoma development. Gut (2020) 69:1841–54. doi: 10.1136/gutjnl-2019-319019 31919231

[12] Shen T Lu Y Zhang Q. High squalene epoxidase in tumors predicts worse survival in patients with hepatocellular carcinoma: Integrated bioinformatic analysis on NAFLD and HCC. Cancer Control (2020) 27:1073274820914663. doi: 10.1177/1073274820914663 32216563 PMC7137641

[13] Song D Wang Y Zhu K Tian L Gao Q Zhou J. DCK is a promising prognostic biomarker and correlated with immune infiltrates in hepatocellular carcinoma. World J Surg Oncol (2020) 18:176. doi: 10.1186/s12957-020-01953-1 32690026 PMC7372783

[14] Toyoda Y Hagiya Y Adachi T Hoshijima K Kuo MT Ishikawa T. MRP class of human ATP binding cassette (ABC) transporters: historical background and new research directions. Xenobiotica (2008) 38:833–62. doi: 10.1080/00498250701883514 18668432

[15] Robey RW Pluchino KM Hall MD Fojo AT Bates SE Gottesman MM. Revisiting the role of ABC transporters in multidrug-resistant cancer. Nat Rev Cancer (2018) 18:452–64. doi: 10.1038/s41568-018-0005-8 PMC6622180 29643473

[16] Hardwick RN Fisher CD Canet MJ Scheffer GL Cherrington NJ. Variations in ATP-binding cassette transporter regulation during the progression of human nonalcoholic fatty liver disease. Drug Metab Dispos (2011) 39:2395–402. doi: 10.1124/dmd.111.041012 PMC3226375 21878559

[17] Davis S Meltzer PS. GEOquery: a bridge between the gene expression omnibus (GEO) and BioConductor. Bioinformatics (2007) 23:1846–7. doi: 10.1093/bioinformatics/btm254 17496320

[18] Ahrens M Ammerpohl O von Schönfels W Kolarova J Bens S Itzel T. DNA Methylation analysis in nonalcoholic fatty liver disease suggests distinct disease-specific and remodeling signatures after bariatric surgery. Cell Metab (2013) 18:296–302. doi: 10.1016/j.cmet.2013.07.004 23931760

[19] Arendt BM Comelli EM Ma DW Lou W Teterina A Kim T. Altered hepatic gene expression in nonalcoholic fatty liver disease is associated with lower hepatic n-3 and n-6 polyunsaturated fatty acids. Hepatology (2015) 61:1565–78. doi: 10.1002/hep.27695 25581263

[20] López-Vicario C González-Périz A Rius B Morán-Salvador E García-Alonso V Lozano JJ. Molecular interplay between Δ5/Δ6 desaturases and long-chain fatty acids in the pathogenesis of non-alcoholic steatohepatitis. Gut (2014) 63:344–55. doi: 10.1136/gutjnl-2012-303179 23492103

[21] Leek JT Johnson WE Parker HS Jaffe AE Storey JD. The sva package for removing batch effects and other unwanted variation in high-throughput experiments. Bioinformatics (2012) 28:882–3. doi: 10.1093/bioinformatics/bts034 PMC3307112 22257669

[22] Ritchie ME Phipson B Wu D Hu Y Law CW Shi W. Limma powers differential expression analyses for RNA-sequencing and microarray studies. Nucleic Acids Res (2015) 43:e47. doi: 10.1093/nar/gkv007 25605792 PMC4402510

[23] Colaprico A Silva TC Olsen C Garofano L Cava C Garolini D. TCGAbiolinks: an R/Bioconductor package for integrative analysis of TCGA data. Nucleic Acids Res (2016) 44:e71. doi: 10.1093/nar/gkv1507 26704973 PMC4856967

[24] Friedman JH Hastie T Tibshirani R. Regularization paths for generalized linear models *via* coordinate descent. J Stat Software (2010) 33:1–22. doi: 10.18637/jss.v033.i01 PMC2929880 20808728

[25] Love MI Huber W Anders S. Moderated estimation of fold change and dispersion for RNA-seq data with DESeq2. Genome Biol (2014) 15:550. doi: 10.1186/s13059-014-0550-8 25516281 PMC4302049

[26] Wickham H (2016). ggplot2: Elegant Graphics for Data Analysis. Springer-Verlag New York. https://ggplot2.tidyverse.org.

[27] Yu G Wang LG Han Y He QY. clusterProfiler: an r package for comparing biological themes among gene clusters. OMICS (2012) 16:284–7. doi: 10.1089/omi.2011.0118 PMC3339379 22455463

[28] Hänzelmann S Castelo R Guinney J. GSVA: gene set variation analysis for microarray and RNA-seq data. BMC Bioinf (2013) 14:7. doi: 10.1186/1471-2105-14-7 PMC3618321 23323831

[29] Langfelder P Horvath S. WGCNA: an r package for weighted correlation network analysis. BMC Bioinf (2008) 9:559. doi: 10.1186/1471-2105-9-559 PMC2631488 19114008

[30] Ru Y Kechris KJ Tabakoff B Hoffman P Radcliffe RA Bowler R. The multiMiR r package and database: integration of microRNA-target interactions along with their disease and drug associations. Nucleic Acids Res (2014) 42:e133. doi: 10.1093/nar/gku631 25063298 PMC4176155

[31] Zhou X Huang JM Li TM Liu JQ Wei ZL Lan CL. Clinical significance and potential mechanisms of ATP binding cassette subfamily c genes in hepatocellular carcinoma. Front Genet (2022) 13:805961. doi: 10.3389/fgene.2022.805961 35342392 PMC8948437

[32] Bozal-Basterra L Gonzalez-Santamarta M Muratore V Martín-Martín N Ercilla A Rodríguez JA. LUZP1 controls cell division, migration and invasion through regulation of the actin cytoskeleton. Front Cell Dev Biol (2021) 9:624089. doi: 10.3389/fcell.2021.624089 33869174 PMC8049182

[33] Chen S Che S Li S Ruan Z. The combined impact of decabromodiphenyl ether and high fat exposure on non-alcoholic fatty liver disease *in vivo* and in vitro. Toxicology (2021) 464:153015. doi: 10.1016/j.tox.2021.153015 34757160

[34] Qiao JT Cui C Qing L Wang LS He TY Yan F. Activation of the STING-IRF3 pathway promotes hepatocyte inflammation, apoptosis and induces metabolic disorders in nonalcoholic fatty liver disease. Metabolism (2018) 81:13–24. doi: 10.1016/j.metabol.2017.09.010 29106945

[35] Zheng Z Li Y Fan S An J Luo X Liang M. WW domain-binding protein 2 overexpression prevents diet-induced liver steatosis and insulin resistance through AMPKβ1. Cell Death Dis (2021) 12:228. doi: 10.1038/s41419-021-03536-8 33658485 PMC7930037

[36] Zhang X Lin Y Lin S Li C Gao J Feng Z. Silencing of functional p53 attenuates NAFLD by promoting HMGB1-related autophagy induction. Hepatol Int (2020) 14:828–41. doi: 10.1007/s12072-020-10068-4 PMC7561543 32607732

[37] Wong AM Ding X Wong AM Xu M. Zhang L. Leung H.H.. Unique molecular characteristics of NAFLD-associated liver cancer accentuate β-catenin/TNFRSF19-mediated immune evasion. J Hepatol (2022) 77(2):410–23. doi: 10.1016/j.jhep.2022.03.015 35351523

[38] Zhang X Coker OO Chu ES Fu K Lau HCH Wang YX. Dietary cholesterol drives fatty liver-associated liver cancer by modulating gut microbiota and metabolites. Gut (2021) 70:761–74. doi: 10.1136/gutjnl-2019-319664 PMC7948195 32694178

[39] Liu D Wong CC Fu L Chen H Zhao L Li C. Squalene epoxidase drives NAFLD-induced hepatocellular carcinoma and is a pharmaceutical target. Sci Transl Med (2018) 10(437):eaap9840. doi: 10.1126/scitranslmed.aap9840 29669855

[40] Borel F Han R Visser A Petry H van Deventer SJ Jansen PL. Adenosine triphosphate-binding cassette transporter genes up-regulation in untreated hepatocellular carcinoma is mediated by cellular microRNAs. Hepatology (2012) 55:821–32. doi: 10.1002/hep.24682 21932399

[41] Chen L Yang Z Cao Y Hu Y Bao W Wu D. Pan-cancer analysis and single-cell analysis revealed the role of ABCC5 transporter in hepatocellular carcinoma. Channels (Austin Tex) (2021) 15:541–54. doi: 10.1080/19336950.2021.1968592 PMC8437464 34494510

[42] Huang W Chen K Lu Y Zhang D Cheng Y Li L. ABCC5 facilitates the acquired resistance of sorafenib through the inhibition of SLC7A11-induced ferroptosis in hepatocellular carcinoma. Neoplasia (New York NY) (2021) 23:1227–39. doi: 10.1016/j.neo.2021.11.002 PMC8591347 34768109

[43] Jedlitschky G Burchell B Keppler D. The multidrug resistance protein 5 functions as an ATP-dependent export pump for cyclic nucleotides. J Biol Chem (2000) 275:30069–74. doi: 10.1074/jbc.M005463200 10893247

[44] Kwong E Li Y Hylemon PB Zhou H. Bile acids and sphingosine-1-phosphate receptor 2 in hepatic lipid metabolism. Acta Pharm Sin B (2015) 5:151–7. doi: 10.1016/j.apsb.2014.12.009 PMC4629213 26579441

[45] Lyu J Imachi H Fukunaga K Sato S Kobayashi T Dong T. Role of ATP-binding cassette transporter A1 in suppressing lipid accumulation by glucagon-like peptide-1 agonist in hepatocytes. Mol Metab (2020) 34:16–26. doi: 10.1016/j.molmet.2019.12.015 32180556 PMC6997505

[46] Liu W Qin L Yu H Lv F Wang Y. Apolipoprotein a-I and adenosine triphosphate-binding cassette transporter A1 expression alleviates lipid accumulation in hepatocytes. J Gastroenterol Hepatol (2014) 29:614–22. doi: 10.1111/jgh.12430 24219083

[47] Akbulut UE Emeksiz HC Citli S Cebi AH Korkmaz HAA Baki G. IL-17A, MCP-1, CCR-2, and ABCA1 polymorphisms in children with non-alcoholic fatty liver disease. J Pediatr (Rio J) (2019) 95:350–7. doi: 10.1016/j.jped.2018.03.005 29733805

[48] Cyranka M Veprik A McKay EJ van Loon N Thijsse A Cotter L. Abcc5 knockout mice have lower fat mass and increased levels of circulating GLP-1. Obes (Silver Spring Md) (2019) 27:1292–304. doi: 10.1002/oby.22521 PMC6658130 31338999

[49] Hubert T Vandekerckhove J Gettemans J. Cdk1 and BRCA1 target γ-tubulin to microtubule domains. Biochem Biophys Res Commun (2011) 414:240–5. doi: 10.1016/j.bbrc.2011.09.064 21951856

[50] Hsu LC White RL. BRCA1 is associated with the centrosome during mitosis. Proc Natl Acad Sci U S A (1998) 95:12983–8. doi: 10.1073/pnas.95.22.12983 PMC23679 9789027

[51] Morris VB Brammall J Noble J Reddel R. p53 localizes to the centrosomes and spindles of mitotic cells in the embryonic chick epiblast, human cell lines, and a human primary culture: An immunofluorescence study. Exp Cell Res (2000) 256:122–30. doi: 10.1006/excr.2000.4800 10739659

[52] Kanai M Tong WM Sugihara E Wang ZQ Fukasawa K Miwa M. Involvement of poly(ADP-ribose) polymerase 1 and poly(ADP-ribosyl)ation in regulation of centrosome function. Mol Cell Biol (2003) 23:2451–62. doi: 10.1128/MCB.23.7.2451-2462.2003 PMC150716 12640128

[53] Hořejší B Vinopal S Sládková V Dráberová E Sulimenko V Sulimenko T. Nuclear γ-tubulin associates with nucleoli and interacts with tumor suppressor protein C53. J Cell Physiol (2012) 227:367–82. doi: 10.1002/jcp.22772 21465471

[54] Olson JE Wang X Pankratz VS Fredericksen ZS Vachon CM Vierkant RA. Centrosome-related genes, genetic variation, and risk of breast cancer. Breast Cancer Res Treat (2011) 125:221–8. doi: 10.1007/s10549-010-0950-8 PMC2997159 20508983

[55] Blanco I Kuchenbaecker K Cuadras D Wang X Barrowdale D de Garibay GR. Assessing associations between the AURKA-HMMR-TPX2-TUBG1 functional module and breast cancer risk in BRCA1/2 mutation carriers. PloS One (2015) 10:e0120020. doi: 10.1371/journal.pone.0120020 25830658 PMC4382299

[56] Chao Y Zhou D. lncRNA-D16366 is a potential biomarker for diagnosis and prognosis of hepatocellular carcinoma. Med Sci Monit (2019) 25:6581–6. doi: 10.12659/MSM.915100 PMC6738002 31475695

[57] Zhou C Li R Mi W. circ_0067934: A potential biomarker and therapeutic target for hepatocellular carcinoma. Ann Clin Lab Sci (2020) 50:734–8. 33334787

