# Supplementary figures and images for: Screening of co-pathogenic genes of non-alcoholic fatty liver disease and hepatocellular carcinoma
Source: Front Oncol. 2022 Aug 11;12:911808. doi: 10.3389/fonc.2022.911808 (PMC9410624; doi:10.3389/fonc.2022.911808)

## Supplement Figure 1

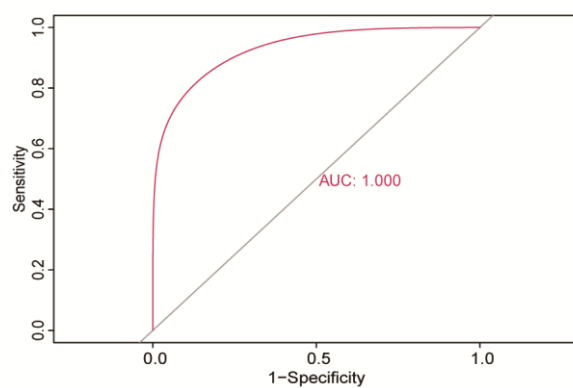

The ROC curve of risk score based on GSE37031

Supplement: Supplementary Figure 1 — The ROC curve of risk score based on GSE37031 [file Image_1.pdf]
