# Supplementary material for: Screening of co-pathogenic genes of non-alcoholic fatty liver disease and hepatocellular carcinoma
Source: Front Oncol. 2022 Aug 11;12:911808. doi: 10.3389/fonc.2022.911808 (PMC9410624; doi:10.3389/fonc.2022.911808)
Supplement: Supplementary file 2 [file Table_1.docx]

**Supplement Table 1 GO function analysis of genes co-regulated in both disease**

| **ID** | **Description** | **p.adjust** |
| --- | --- | --- |
| GO:0032496 | response to lipopolysaccharide | 1.85E-09 |
| GO:0002237 | response to molecule of bacterial origin | 1.85E-09 |
| GO:0150076 | neuroinflammatory response | 5.15E-07 |
| GO:0048545 | response to steroid hormone | 5.38E-07 |
| GO:0060326 | cell chemotaxis | 1.58E-06 |
| GO:0042116 | macrophage activation | 2.19E-06 |
| GO:0048661 | positive regulation of smooth muscle cell proliferation | 3.24E-06 |
| GO:0051384 | response to glucocorticoid | 5.03E-06 |
| GO:0033002 | muscle cell proliferation | 5.03E-06 |
| GO:0048511 | rhythmic process | 5.17E-06 |
| GO:0050673 | epithelial cell proliferation | 8.89E-06 |
| GO:0031960 | response to corticosteroid | 9.31E-06 |
| GO:0050678 | regulation of epithelial cell proliferation | 9.59E-06 |
| GO:0061900 | glial cell activation | 9.81E-06 |
| GO:0048660 | regulation of smooth muscle cell proliferation | 1.11E-05 |
| GO:0048659 | smooth muscle cell proliferation | 1.17E-05 |
| GO:0030595 | leukocyte chemotaxis | 1.39E-05 |
| GO:0019216 | regulation of lipid metabolic process | 1.89E-05 |
| GO:0045834 | positive regulation of lipid metabolic process | 2.98E-05 |
| GO:0014002 | astrocyte development | 3.14E-05 |
| GO:0031100 | animal organ regeneration | 3.20E-05 |
| GO:0031099 | regeneration | 3.20E-05 |
| GO:0046890 | regulation of lipid biosynthetic process | 3.20E-05 |
| GO:0050729 | positive regulation of inflammatory response | 3.40E-05 |
| GO:1904019 | epithelial cell apoptotic process | 3.40E-05 |
| GO:0071222 | cellular response to lipopolysaccharide | 3.82E-05 |
| GO:0001774 | microglial cell activation | 3.82E-05 |
| GO:0002269 | leukocyte activation involved in inflammatory response | 3.82E-05 |
| GO:0046886 | positive regulation of hormone biosynthetic process | 3.82E-05 |
| GO:0007623 | circadian rhythm | 3.87E-05 |
| GO:0097529 | myeloid leukocyte migration | 4.10E-05 |
| GO:0071219 | cellular response to molecule of bacterial origin | 4.26E-05 |
| GO:0048143 | astrocyte activation | 4.26E-05 |
| GO:0070486 | leukocyte aggregation | 4.86E-05 |
| GO:0046889 | positive regulation of lipid biosynthetic process | 5.23E-05 |
| GO:0044706 | multi-multicellular organism process | 5.87E-05 |
| GO:0045444 | fat cell differentiation | 5.96E-05 |
| GO:2000182 | regulation of progesterone biosynthetic process | 6.45E-05 |
| GO:1904035 | regulation of epithelial cell apoptotic process | 6.45E-05 |
| GO:0032602 | chemokine production | 6.79E-05 |
| GO:0071216 | cellular response to biotic stimulus | 9.00E-05 |
| GO:0032352 | positive regulation of hormone metabolic process | 0.000107 |
| GO:0062013 | positive regulation of small molecule metabolic process | 0.000122 |
| GO:0042180 | cellular ketone metabolic process | 0.000122 |
| GO:0050727 | regulation of inflammatory response | 0.000125 |
| GO:0042448 | progesterone metabolic process | 0.000125 |
| GO:0043435 | response to corticotropin-releasing hormone | 0.000125 |
| GO:0070487 | monocyte aggregation | 0.000125 |
| GO:0071376 | cellular response to corticotropin-releasing hormone stimulus | 0.000125 |
| GO:0045923 | positive regulation of fatty acid metabolic process | 0.000145 |
| GO:0014074 | response to purine-containing compound | 0.000157 |
| GO:0001819 | positive regulation of cytokine production | 0.000177 |
| GO:0031394 | positive regulation of prostaglandin biosynthetic process | 0.000227 |
| GO:0150077 | regulation of neuroinflammatory response | 0.000227 |
| GO:0070997 | neuron death | 0.00033 |
| GO:0032570 | response to progesterone | 0.00035 |
| GO:0031667 | response to nutrient levels | 0.000355 |
| GO:0006701 | progesterone biosynthetic process | 0.000365 |
| GO:0062012 | regulation of small molecule metabolic process | 0.000392 |
| GO:0071621 | granulocyte chemotaxis | 0.000397 |
| GO:0048708 | astrocyte differentiation | 0.000398 |
| GO:0032642 | regulation of chemokine production | 0.000421 |
| GO:0014910 | regulation of smooth muscle cell migration | 0.000476 |
| GO:0010565 | regulation of cellular ketone metabolic process | 0.000506 |
| GO:2001280 | positive regulation of unsaturated fatty acid biosynthetic process | 0.000519 |
| GO:0070542 | response to fatty acid | 0.000521 |
| GO:0046683 | response to organophosphorus | 0.000624 |
| GO:0071248 | cellular response to metal ion | 0.000644 |
| GO:0071674 | mononuclear cell migration | 0.000644 |
| GO:1901214 | regulation of neuron death | 0.000644 |
| GO:0014909 | smooth muscle cell migration | 0.000671 |
| GO:0007565 | female pregnancy | 0.000718 |
| GO:0043030 | regulation of macrophage activation | 0.000726 |
| GO:1901654 | response to ketone | 0.000726 |
| GO:0046885 | regulation of hormone biosynthetic process | 0.000731 |
| GO:0097530 | granulocyte migration | 0.000767 |
| GO:0019217 | regulation of fatty acid metabolic process | 0.000841 |
| GO:0032102 | negative regulation of response to external stimulus | 0.000856 |
| GO:0022407 | regulation of cell-cell adhesion | 0.000885 |
| GO:0010575 | positive regulation of vascular endothelial growth factor production | 0.000919 |
| GO:0031668 | cellular response to extracellular stimulus | 0.001034 |
| GO:0007159 | leukocyte cell-cell adhesion | 0.001034 |
| GO:0050679 | positive regulation of epithelial cell proliferation | 0.001034 |
| GO:0071496 | cellular response to external stimulus | 0.001064 |
| GO:1901215 | negative regulation of neuron death | 0.001082 |
| GO:0000187 | activation of MAPK activity | 0.0011 |
| GO:0009612 | response to mechanical stimulus | 0.001123 |
| GO:0001101 | response to acid chemical | 0.001123 |
| GO:0014812 | muscle cell migration | 0.001134 |
| GO:0030593 | neutrophil chemotaxis | 0.001134 |
| GO:0050900 | leukocyte migration | 0.001139 |
| GO:2000184 | positive regulation of progesterone biosynthetic process | 0.001172 |
| GO:1901342 | regulation of vasculature development | 0.001172 |
| GO:0071241 | cellular response to inorganic substance | 0.001234 |
| GO:0062197 | cellular response to chemical stress | 0.001234 |
| GO:0002526 | acute inflammatory response | 0.001316 |
| GO:0031392 | regulation of prostaglandin biosynthetic process | 0.001344 |
| GO:0051090 | regulation of DNA-binding transcription factor activity | 0.001376 |
| GO:0010574 | regulation of vascular endothelial growth factor production | 0.001419 |
| GO:0002683 | negative regulation of immune system process | 0.001436 |
| GO:0043434 | response to peptide hormone | 0.001451 |
| GO:0007162 | negative regulation of cell adhesion | 0.001481 |
| GO:0010038 | response to metal ion | 0.001512 |
| GO:0002430 | complement receptor mediated signaling pathway | 0.0016 |
| GO:0061469 | regulation of type B pancreatic cell proliferation | 0.0016 |
| GO:0002688 | regulation of leukocyte chemotaxis | 0.0016 |
| GO:0042035 | regulation of cytokine biosynthetic process | 0.0016 |
| GO:0010573 | vascular endothelial growth factor production | 0.001637 |
| GO:0036293 | response to decreased oxygen levels | 0.001685 |
| GO:0009743 | response to carbohydrate | 0.001685 |
| GO:1904018 | positive regulation of vasculature development | 0.001685 |
| GO:0021782 | glial cell development | 0.001685 |
| GO:0030212 | hyaluronan metabolic process | 0.001743 |
| GO:0006979 | response to oxidative stress | 0.001743 |
| GO:0097305 | response to alcohol | 0.001785 |
| GO:1990266 | neutrophil migration | 0.001785 |
| GO:0030213 | hyaluronan biosynthetic process | 0.001785 |
| GO:0034356 | NAD biosynthesis via nicotinamide riboside salvage pathway | 0.001785 |
| GO:2001279 | regulation of unsaturated fatty acid biosynthetic process | 0.001785 |
| GO:0001659 | temperature homeostasis | 0.001785 |
| GO:0032350 | regulation of hormone metabolic process | 0.001785 |
| GO:0048246 | macrophage chemotaxis | 0.001785 |
| GO:1904037 | positive regulation of epithelial cell apoptotic process | 0.001785 |
| GO:1903037 | regulation of leukocyte cell-cell adhesion | 0.001799 |
| GO:0008207 | C21-steroid hormone metabolic process | 0.001949 |
| GO:0045765 | regulation of angiogenesis | 0.001978 |
| GO:0031349 | positive regulation of defense response | 0.002005 |
| GO:0042089 | cytokine biosynthetic process | 0.002044 |
| GO:0045073 | regulation of chemokine biosynthetic process | 0.002063 |
| GO:0019218 | regulation of steroid metabolic process | 0.002088 |
| GO:0042107 | cytokine metabolic process | 0.002088 |
| GO:0050873 | brown fat cell differentiation | 0.002252 |
| GO:0070371 | ERK1 and ERK2 cascade | 0.002309 |
| GO:0001570 | vasculogenesis | 0.002309 |
| GO:0070482 | response to oxygen levels | 0.002314 |
| GO:0035633 | maintenance of blood-brain barrier | 0.002314 |
| GO:1900623 | regulation of monocyte aggregation | 0.002314 |
| GO:1900625 | positive regulation of monocyte aggregation | 0.002314 |
| GO:0010566 | regulation of ketone biosynthetic process | 0.002314 |
| GO:0042033 | chemokine biosynthetic process | 0.002314 |
| GO:0050755 | chemokine metabolic process | 0.002314 |
| GO:0007568 | aging | 0.002379 |
| GO:0045785 | positive regulation of cell adhesion | 0.00265 |
| GO:0022409 | positive regulation of cell-cell adhesion | 0.002661 |
| GO:0048871 | multicellular organismal homeostasis | 0.002661 |
| GO:0046677 | response to antibiotic | 0.002662 |
| GO:0001935 | endothelial cell proliferation | 0.002709 |
| GO:0042594 | response to starvation | 0.002709 |
| GO:0043406 | positive regulation of MAP kinase activity | 0.002801 |
| GO:0071675 | regulation of mononuclear cell migration | 0.003122 |
| GO:0150078 | positive regulation of neuroinflammatory response | 0.003122 |
| GO:0043405 | regulation of MAP kinase activity | 0.003188 |
| GO:0002690 | positive regulation of leukocyte chemotaxis | 0.003188 |
| GO:0030099 | myeloid cell differentiation | 0.003188 |
| GO:0006953 | acute-phase response | 0.003289 |
| GO:0002544 | chronic inflammatory response | 0.003573 |
| GO:1904707 | positive regulation of vascular smooth muscle cell proliferation | 0.003811 |
| GO:0045766 | positive regulation of angiogenesis | 0.003811 |
| GO:1903204 | negative regulation of oxidative stress-induced neuron death | 0.004107 |
| GO:0042542 | response to hydrogen peroxide | 0.00419 |
| GO:0043524 | negative regulation of neuron apoptotic process | 0.004321 |
| GO:0043523 | regulation of neuron apoptotic process | 0.004439 |
| GO:0050810 | regulation of steroid biosynthetic process | 0.004516 |
| GO:0010893 | positive regulation of steroid biosynthetic process | 0.004516 |
| GO:0030728 | ovulation | 0.004516 |
| GO:0032682 | negative regulation of chemokine production | 0.004516 |
| GO:0036499 | PERK-mediated unfolded protein response | 0.004516 |
| GO:0051412 | response to corticosterone | 0.004516 |
| GO:0001666 | response to hypoxia | 0.004619 |
| GO:1905517 | macrophage migration | 0.004794 |
| GO:0044342 | type B pancreatic cell proliferation | 0.005086 |
| GO:0045723 | positive regulation of fatty acid biosynthetic process | 0.005086 |
| GO:0010001 | glial cell differentiation | 0.00519 |
| GO:1903039 | positive regulation of leukocyte cell-cell adhesion | 0.00519 |
| GO:0042063 | gliogenesis | 0.005285 |
| GO:0042304 | regulation of fatty acid biosynthetic process | 0.005346 |
| GO:0034695 | response to prostaglandin E | 0.005659 |
| GO:0036476 | neuron death in response to hydrogen peroxide | 0.00592 |
| GO:1903207 | regulation of hydrogen peroxide-induced neuron death | 0.00592 |
| GO:1903208 | negative regulation of hydrogen peroxide-induced neuron death | 0.00592 |
| GO:2000660 | negative regulation of interleukin-1-mediated signaling pathway | 0.00592 |
| GO:0071398 | cellular response to fatty acid | 0.005934 |
| GO:0030203 | glycosaminoglycan metabolic process | 0.006 |
| GO:0032722 | positive regulation of chemokine production | 0.006273 |
| GO:0070372 | regulation of ERK1 and ERK2 cascade | 0.006273 |
| GO:0002696 | positive regulation of leukocyte activation | 0.006368 |
| GO:0034599 | cellular response to oxidative stress | 0.006487 |
| GO:0042326 | negative regulation of phosphorylation | 0.006692 |
| GO:1901653 | cellular response to peptide | 0.006892 |
| GO:0000302 | response to reactive oxygen species | 0.006921 |
| GO:0006700 | C21-steroid hormone biosynthetic process | 0.007478 |
| GO:0031645 | negative regulation of nervous system process | 0.007478 |
| GO:0034114 | regulation of heterotypic cell-cell adhesion | 0.007478 |
| GO:1903203 | regulation of oxidative stress-induced neuron death | 0.007478 |
| GO:0006024 | glycosaminoglycan biosynthetic process | 0.007478 |
| GO:0006022 | aminoglycan metabolic process | 0.007737 |
| GO:0050867 | positive regulation of cell activation | 0.007816 |
| GO:0051402 | neuron apoptotic process | 0.007934 |
| GO:0010757 | negative regulation of plasminogen activation | 0.008047 |
| GO:0036475 | neuron death in response to oxidative stress | 0.008094 |
| GO:0043032 | positive regulation of macrophage activation | 0.008094 |
| GO:0007204 | positive regulation of cytosolic calcium ion concentration | 0.008629 |
| GO:0002548 | monocyte chemotaxis | 0.008674 |
| GO:0072577 | endothelial cell apoptotic process | 0.008674 |
| GO:0006023 | aminoglycan biosynthetic process | 0.008674 |
| GO:0001516 | prostaglandin biosynthetic process | 0.008674 |
| GO:0042730 | fibrinolysis | 0.008674 |
| GO:0046457 | prostanoid biosynthetic process | 0.008674 |
| GO:2000108 | positive regulation of leukocyte apoptotic process | 0.008674 |
| GO:0001936 | regulation of endothelial cell proliferation | 0.008674 |
| GO:0072503 | cellular divalent inorganic cation homeostasis | 0.008946 |
| GO:0097237 | cellular response to toxic substance | 0.009012 |
| GO:0060537 | muscle tissue development | 0.009287 |
| GO:0071347 | cellular response to interleukin-1 | 0.009287 |
| GO:0042108 | positive regulation of cytokine biosynthetic process | 0.009287 |
| GO:0007263 | nitric oxide mediated signal transduction | 0.009287 |
| GO:0009435 | NAD biosynthetic process | 0.009287 |
| GO:0007517 | muscle organ development | 0.009397 |
| GO:0035914 | skeletal muscle cell differentiation | 0.009684 |
| GO:0008202 | steroid metabolic process | 0.009962 |
| GO:0045940 | positive regulation of steroid metabolic process | 0.009962 |
| GO:0031622 | positive regulation of fever generation | 0.009962 |
| GO:2000659 | regulation of interleukin-1-mediated signaling pathway | 0.009962 |
| GO:0002040 | sprouting angiogenesis | 0.009962 |
| GO:0042698 | ovulation cycle | 0.009962 |
| GO:0032147 | activation of protein kinase activity | 0.010215 |
| GO:0071902 | positive regulation of protein serine/threonine kinase activity | 0.01037 |
| GO:0002675 | positive regulation of acute inflammatory response | 0.010689 |
| GO:0034694 | response to prostaglandin | 0.010689 |
| GO:0035767 | endothelial cell chemotaxis | 0.010689 |
| GO:0019359 | nicotinamide nucleotide biosynthetic process | 0.011544 |
| GO:0019363 | pyridine nucleotide biosynthetic process | 0.011544 |
| GO:0051385 | response to mineralocorticoid | 0.011544 |
| GO:0051968 | positive regulation of synaptic transmission, glutamatergic | 0.011544 |
| GO:1903524 | positive regulation of blood circulation | 0.011772 |
| GO:0051047 | positive regulation of secretion | 0.011772 |
| GO:0001933 | negative regulation of protein phosphorylation | 0.011917 |
| GO:0002687 | positive regulation of leukocyte migration | 0.01201 |
| GO:0010955 | negative regulation of protein processing | 0.01201 |
| GO:0019362 | pyridine nucleotide metabolic process | 0.01201 |
| GO:0046496 | nicotinamide nucleotide metabolic process | 0.01201 |
| GO:1903318 | negative regulation of protein maturation | 0.01201 |
| GO:2000758 | positive regulation of peptidyl-lysine acetylation | 0.01201 |
| GO:0009750 | response to fructose | 0.01201 |
| GO:0031620 | regulation of fever generation | 0.01201 |
| GO:0061888 | regulation of astrocyte activation | 0.01201 |
| GO:1901724 | positive regulation of cell proliferation involved in kidney development | 0.01201 |
| GO:0034754 | cellular hormone metabolic process | 0.012107 |
| GO:0035690 | cellular response to drug | 0.012107 |
| GO:0002685 | regulation of leukocyte migration | 0.012727 |
| GO:0006694 | steroid biosynthetic process | 0.012727 |
| GO:0097009 | energy homeostasis | 0.012825 |
| GO:0010631 | epithelial cell migration | 0.013059 |
| GO:0035966 | response to topologically incorrect protein | 0.013603 |
| GO:0090132 | epithelium migration | 0.013681 |
| GO:0032689 | negative regulation of interferon-gamma production | 0.013697 |
| GO:0072525 | pyridine-containing compound biosynthetic process | 0.013697 |
| GO:0051480 | regulation of cytosolic calcium ion concentration | 0.014016 |
| GO:0045861 | negative regulation of proteolysis | 0.014177 |
| GO:0050921 | positive regulation of chemotaxis | 0.014177 |
| GO:0009746 | response to hexose | 0.014298 |
| GO:0001542 | ovulation from ovarian follicle | 0.014503 |
| GO:0009165 | nucleotide biosynthetic process | 0.014506 |
| GO:0090130 | tissue migration | 0.014707 |
| GO:1901293 | nucleoside phosphate biosynthetic process | 0.015579 |
| GO:0014912 | negative regulation of smooth muscle cell migration | 0.015579 |
| GO:0050715 | positive regulation of cytokine secretion | 0.015721 |
| GO:0034284 | response to monosaccharide | 0.015725 |
| GO:0070555 | response to interleukin-1 | 0.015725 |
| GO:0034976 | response to endoplasmic reticulum stress | 0.016304 |
| GO:0071229 | cellular response to acid chemical | 0.016395 |
| GO:0006970 | response to osmotic stress | 0.016405 |
| GO:0071277 | cellular response to calcium ion | 0.016405 |
| GO:0002793 | positive regulation of peptide secretion | 0.016899 |
| GO:0002718 | regulation of cytokine production involved in immune response | 0.016899 |
| GO:0001667 | ameboidal-type cell migration | 0.016899 |
| GO:0001660 | fever generation | 0.016899 |
| GO:0051918 | negative regulation of fibrinolysis | 0.016899 |
| GO:0072203 | cell proliferation involved in metanephros development | 0.016899 |
| GO:0106049 | regulation of cellular response to osmotic stress | 0.016899 |
| GO:0044344 | cellular response to fibroblast growth factor stimulus | 0.016939 |
| GO:0072524 | pyridine-containing compound metabolic process | 0.01714 |
| GO:0042110 | T cell activation | 0.017163 |
| GO:1904705 | regulation of vascular smooth muscle cell proliferation | 0.017163 |
| GO:1990874 | vascular smooth muscle cell proliferation | 0.017163 |
| GO:0002822 | regulation of adaptive immune response based on somatic recombination of immune receptors built from immunoglobulin superfamily domains | 0.017695 |
| GO:0042446 | hormone biosynthetic process | 0.017796 |
| GO:0042036 | negative regulation of cytokine biosynthetic process | 0.018067 |
| GO:0050920 | regulation of chemotaxis | 0.018609 |
| GO:0001818 | negative regulation of cytokine production | 0.018779 |
| GO:0050890 | cognition | 0.018779 |
| GO:0051592 | response to calcium ion | 0.018968 |
| GO:0000727 | double-strand break repair via break-induced replication | 0.019398 |
| GO:0031652 | positive regulation of heat generation | 0.019398 |
| GO:0032494 | response to peptidoglycan | 0.019398 |
| GO:0034115 | negative regulation of heterotypic cell-cell adhesion | 0.019398 |
| GO:0070587 | regulation of cell-cell adhesion involved in gastrulation | 0.019398 |
| GO:0006090 | pyruvate metabolic process | 0.019648 |
| GO:0071774 | response to fibroblast growth factor | 0.019648 |
| GO:0042181 | ketone biosynthetic process | 0.019985 |
| GO:1903035 | negative regulation of response to wounding | 0.02006 |
| GO:0001890 | placenta development | 0.020615 |
| GO:0014706 | striated muscle tissue development | 0.020848 |
| GO:0006692 | prostanoid metabolic process | 0.020907 |
| GO:0006693 | prostaglandin metabolic process | 0.020907 |
| GO:0042088 | T-helper 1 type immune response | 0.020907 |
| GO:1901985 | positive regulation of protein acetylation | 0.020907 |
| GO:0030950 | establishment or maintenance of actin cytoskeleton polarity | 0.022146 |
| GO:0045080 | positive regulation of chemokine biosynthetic process | 0.022146 |
| GO:0060670 | branching involved in labyrinthine layer morphogenesis | 0.022146 |
| GO:0070586 | cell-cell adhesion involved in gastrulation | 0.022146 |
| GO:0042129 | regulation of T cell proliferation | 0.022351 |
| GO:0034612 | response to tumor necrosis factor | 0.023501 |
| GO:0050863 | regulation of T cell activation | 0.02428 |
| GO:0042445 | hormone metabolic process | 0.024288 |
| GO:1903532 | positive regulation of secretion by cell | 0.024288 |
| GO:0002819 | regulation of adaptive immune response | 0.024288 |
| GO:0007519 | skeletal muscle tissue development | 0.024288 |
| GO:0002673 | regulation of acute inflammatory response | 0.024288 |
| GO:0006984 | ER-nucleus signaling pathway | 0.024288 |
| GO:0060711 | labyrinthine layer development | 0.024288 |
| GO:0015908 | fatty acid transport | 0.024573 |
| GO:0051591 | response to cAMP | 0.024573 |
| GO:0035967 | cellular response to topologically incorrect protein | 0.024573 |
| GO:2001233 | regulation of apoptotic signaling pathway | 0.024573 |
| GO:0031650 | regulation of heat generation | 0.024573 |
| GO:0045820 | negative regulation of glycolytic process | 0.024573 |
| GO:0047484 | regulation of response to osmotic stress | 0.024573 |
| GO:0060347 | heart trabecula formation | 0.024573 |
| GO:1902947 | regulation of tau-protein kinase activity | 0.024573 |
| GO:0002042 | cell migration involved in sprouting angiogenesis | 0.02483 |
| GO:0014911 | positive regulation of smooth muscle cell migration | 0.02483 |
| GO:0061614 | pri-miRNA transcription by RNA polymerase II | 0.02483 |
| GO:1903580 | positive regulation of ATP metabolic process | 0.02483 |
| GO:0031669 | cellular response to nutrient levels | 0.024985 |
| GO:0070498 | interleukin-1-mediated signaling pathway | 0.02639 |
| GO:0032649 | regulation of interferon-gamma production | 0.027276 |
| GO:0010755 | regulation of plasminogen activation | 0.027558 |
| GO:0051917 | regulation of fibrinolysis | 0.027558 |
| GO:0072216 | positive regulation of metanephros development | 0.027558 |
| GO:1901722 | regulation of cell proliferation involved in kidney development | 0.027558 |
| GO:1902043 | positive regulation of extrinsic apoptotic signaling pathway via death domain receptors | 0.027558 |
| GO:0002367 | cytokine production involved in immune response | 0.027689 |
| GO:2000379 | positive regulation of reactive oxygen species metabolic process | 0.027689 |
| GO:0060538 | skeletal muscle organ development | 0.028577 |
| GO:0042742 | defense response to bacterium | 0.029045 |
| GO:0019233 | sensory perception of pain | 0.029196 |
| GO:0071887 | leukocyte apoptotic process | 0.029196 |
| GO:0071214 | cellular response to abiotic stimulus | 0.029196 |
| GO:0104004 | cellular response to environmental stimulus | 0.029196 |
| GO:0006733 | oxidoreduction coenzyme metabolic process | 0.029842 |
| GO:0006352 | DNA-templated transcription, initiation | 0.030178 |
| GO:0052548 | regulation of endopeptidase activity | 0.030178 |
| GO:0010759 | positive regulation of macrophage chemotaxis | 0.030178 |
| GO:0015671 | oxygen transport | 0.030178 |
| GO:0017014 | protein nitrosylation | 0.030178 |
| GO:0018119 | peptidyl-cysteine S-nitrosylation | 0.030178 |
| GO:0046321 | positive regulation of fatty acid oxidation | 0.030178 |
| GO:0051770 | positive regulation of nitric-oxide synthase biosynthetic process | 0.030178 |
| GO:0010951 | negative regulation of endopeptidase activity | 0.030264 |
| GO:0055078 | sodium ion homeostasis | 0.030663 |
| GO:0045637 | regulation of myeloid cell differentiation | 0.030691 |
| GO:0048608 | reproductive structure development | 0.032013 |
| GO:0030195 | negative regulation of blood coagulation | 0.032013 |
| GO:0038066 | p38MAPK cascade | 0.032013 |
| GO:0006986 | response to unfolded protein | 0.032044 |
| GO:0061458 | reproductive system development | 0.032461 |
| GO:0007611 | learning or memory | 0.032461 |
| GO:0002720 | positive regulation of cytokine production involved in immune response | 0.032461 |
| GO:0006636 | unsaturated fatty acid biosynthetic process | 0.032461 |
| GO:1900047 | negative regulation of hemostasis | 0.032461 |
| GO:0006206 | pyrimidine nucleobase metabolic process | 0.032461 |
| GO:0009130 | pyrimidine nucleoside monophosphate biosynthetic process | 0.032461 |
| GO:0030952 | establishment or maintenance of cytoskeleton polarity | 0.032461 |
| GO:0032354 | response to follicle-stimulating hormone | 0.032461 |
| GO:0032695 | negative regulation of interleukin-12 production | 0.032461 |
| GO:0034116 | positive regulation of heterotypic cell-cell adhesion | 0.032461 |
| GO:0044849 | estrous cycle | 0.032461 |
| GO:0070293 | renal absorption | 0.032461 |
| GO:0071380 | cellular response to prostaglandin E stimulus | 0.032461 |
| GO:1903034 | regulation of response to wounding | 0.033076 |
| GO:0022408 | negative regulation of cell-cell adhesion | 0.033691 |
| GO:0043534 | blood vessel endothelial cell migration | 0.033691 |
| GO:0003007 | heart morphogenesis | 0.033706 |
| GO:0046456 | icosanoid biosynthetic process | 0.033706 |
| GO:0001938 | positive regulation of endothelial cell proliferation | 0.034425 |
| GO:1903510 | mucopolysaccharide metabolic process | 0.034425 |
| GO:0051091 | positive regulation of DNA-binding transcription factor activity | 0.034645 |
| GO:0008217 | regulation of blood pressure | 0.034672 |
| GO:0061138 | morphogenesis of a branching epithelium | 0.034672 |
| GO:0007178 | transmembrane receptor protein serine/threonine kinase signaling pathway | 0.034744 |
| GO:0090183 | regulation of kidney development | 0.034744 |
| GO:1903202 | negative regulation of oxidative stress-induced cell death | 0.034744 |
| GO:0010466 | negative regulation of peptidase activity | 0.034757 |
| GO:0032609 | interferon-gamma production | 0.034812 |
| GO:0009129 | pyrimidine nucleoside monophosphate metabolic process | 0.034818 |
| GO:0031000 | response to caffeine | 0.034818 |
| GO:0031649 | heat generation | 0.034818 |
| GO:0036270 | response to diuretic | 0.034818 |
| GO:0051969 | regulation of transmission of nerve impulse | 0.034818 |
| GO:0042098 | T cell proliferation | 0.035315 |
| GO:0050819 | negative regulation of coagulation | 0.035615 |
| GO:1900408 | negative regulation of cellular response to oxidative stress | 0.035615 |
| GO:0007369 | gastrulation | 0.035864 |
| GO:0032612 | interleukin-1 production | 0.036136 |
| GO:0019229 | regulation of vasoconstriction | 0.036943 |
| GO:0031663 | lipopolysaccharide-mediated signaling pathway | 0.036943 |
| GO:1902041 | regulation of extrinsic apoptotic signaling pathway via death domain receptors | 0.036943 |
| GO:0050714 | positive regulation of protein secretion | 0.037355 |
| GO:0052547 | regulation of peptidase activity | 0.037474 |
| GO:0035994 | response to muscle stretch | 0.037474 |
| GO:0070230 | positive regulation of lymphocyte apoptotic process | 0.037474 |
| GO:0090026 | positive regulation of monocyte chemotaxis | 0.037474 |
| GO:1900543 | negative regulation of purine nucleotide metabolic process | 0.037474 |
| GO:1902001 | fatty acid transmembrane transport | 0.037474 |
| GO:0031102 | neuron projection regeneration | 0.037474 |
| GO:0032890 | regulation of organic acid transport | 0.037474 |
| GO:0042130 | negative regulation of T cell proliferation | 0.037474 |
| GO:0120178 | steroid hormone biosynthetic process | 0.037474 |
| GO:1902883 | negative regulation of response to oxidative stress | 0.037474 |
| GO:2000351 | regulation of endothelial cell apoptotic process | 0.037474 |
| GO:2000756 | regulation of peptidyl-lysine acetylation | 0.037474 |
| GO:0032868 | response to insulin | 0.038787 |
| GO:0031331 | positive regulation of cellular catabolic process | 0.039229 |
| GO:0006874 | cellular calcium ion homeostasis | 0.039418 |
| GO:0003014 | renal system process | 0.039895 |
| GO:0034113 | heterotypic cell-cell adhesion | 0.039895 |
| GO:0002726 | positive regulation of T cell cytokine production | 0.039895 |
| GO:0015669 | gas transport | 0.039895 |
| GO:0045076 | regulation of interleukin-2 biosynthetic process | 0.039895 |
| GO:0045980 | negative regulation of nucleotide metabolic process | 0.039895 |
| GO:0060252 | positive regulation of glial cell proliferation | 0.039895 |
| GO:0030968 | endoplasmic reticulum unfolded protein response | 0.039895 |
| GO:2000377 | regulation of reactive oxygen species metabolic process | 0.039895 |
| GO:0030198 | extracellular matrix organization | 0.039895 |
| GO:0032922 | circadian regulation of gene expression | 0.039895 |
| GO:0001822 | kidney development | 0.039895 |
| GO:0043062 | extracellular structure organization | 0.039895 |
| GO:0001763 | morphogenesis of a branching structure | 0.039895 |
| GO:0000082 | G1/S transition of mitotic cell cycle | 0.039895 |
| GO:0002413 | tolerance induction to tumor cell | 0.039895 |
| GO:0002843 | regulation of tolerance induction to tumor cell | 0.039895 |
| GO:0002845 | positive regulation of tolerance induction to tumor cell | 0.039895 |
| GO:0002875 | negative regulation of chronic inflammatory response to antigenic stimulus | 0.039895 |
| GO:0009444 | pyruvate oxidation | 0.039895 |
| GO:0010335 | response to non-ionic osmotic stress | 0.039895 |
| GO:0035490 | regulation of leukotriene production involved in inflammatory response | 0.039895 |
| GO:0035491 | positive regulation of leukotriene production involved in inflammatory response | 0.039895 |
| GO:0035504 | regulation of myosin light chain kinase activity | 0.039895 |
| GO:0035505 | positive regulation of myosin light chain kinase activity | 0.039895 |
| GO:0038097 | positive regulation of mast cell activation by Fc-epsilon receptor signaling pathway | 0.039895 |
| GO:0045380 | positive regulation of interleukin-17 biosynthetic process | 0.039895 |
| GO:0048688 | negative regulation of sprouting of injured axon | 0.039895 |
| GO:0048692 | negative regulation of axon extension involved in regeneration | 0.039895 |
| GO:0060034 | notochord cell differentiation | 0.039895 |
| GO:0060035 | notochord cell development | 0.039895 |
| GO:0071250 | cellular response to nitrite | 0.039895 |
| GO:0071471 | cellular response to non-ionic osmotic stress | 0.039895 |
| GO:0072303 | positive regulation of glomerular metanephric mesangial cell proliferation | 0.039895 |
| GO:0080033 | response to nitrite | 0.039895 |
| GO:0090095 | regulation of metanephric cap mesenchymal cell proliferation | 0.039895 |
| GO:0090096 | positive regulation of metanephric cap mesenchymal cell proliferation | 0.039895 |
| GO:1901331 | positive regulation of odontoblast differentiation | 0.039895 |
| GO:1901834 | regulation of deadenylation-independent decapping of nuclear-transcribed mRNA | 0.039895 |
| GO:1901835 | positive regulation of deadenylation-independent decapping of nuclear-transcribed mRNA | 0.039895 |
| GO:1901860 | positive regulation of mitochondrial DNA metabolic process | 0.039895 |
| GO:1903210 | glomerular visceral epithelial cell apoptotic process | 0.039895 |
| GO:1904246 | negative regulation of polynucleotide adenylyltransferase activity | 0.039895 |
| GO:1904633 | regulation of glomerular visceral epithelial cell apoptotic process | 0.039895 |
| GO:1904635 | positive regulation of glomerular visceral epithelial cell apoptotic process | 0.039895 |
| GO:1905062 | positive regulation of cardioblast proliferation | 0.039895 |
| GO:1905377 | response to D-galactose | 0.039895 |
| GO:1905404 | positive regulation of activated CD8-positive, alpha-beta T cell apoptotic process | 0.039895 |
| GO:1905603 | regulation of blood-brain barrier permeability | 0.039895 |
| GO:0014013 | regulation of gliogenesis | 0.039997 |
| GO:0045655 | regulation of monocyte differentiation | 0.039997 |
| GO:0051767 | nitric-oxide synthase biosynthetic process | 0.039997 |
| GO:0051769 | regulation of nitric-oxide synthase biosynthetic process | 0.039997 |
| GO:0071636 | positive regulation of transforming growth factor beta production | 0.039997 |
| GO:1903978 | regulation of microglial cell activation | 0.039997 |
| GO:1905523 | positive regulation of macrophage migration | 0.039997 |
| GO:0031032 | actomyosin structure organization | 0.040792 |
| GO:0055074 | calcium ion homeostasis | 0.040879 |
| GO:0043550 | regulation of lipid kinase activity | 0.041012 |
| GO:0003012 | muscle system process | 0.041217 |
| GO:0051348 | negative regulation of transferase activity | 0.042182 |
| GO:0014823 | response to activity | 0.042505 |
| GO:1903706 | regulation of hemopoiesis | 0.042505 |
| GO:0051403 | stress-activated MAPK cascade | 0.042625 |
| GO:0050870 | positive regulation of T cell activation | 0.042694 |
| GO:0035743 | CD4-positive, alpha-beta T cell cytokine production | 0.042882 |
| GO:0060713 | labyrinthine layer morphogenesis | 0.042882 |
| GO:0071498 | cellular response to fluid shear stress | 0.042882 |
| GO:0072111 | cell proliferation involved in kidney development | 0.042882 |
| GO:0045428 | regulation of nitric oxide biosynthetic process | 0.04359 |
| GO:0070613 | regulation of protein processing | 0.04359 |
| GO:0002573 | myeloid leukocyte differentiation | 0.043842 |
| GO:0043500 | muscle adaptation | 0.045154 |
| GO:0048662 | negative regulation of smooth muscle cell proliferation | 0.045154 |
| GO:0010632 | regulation of epithelial cell migration | 0.045154 |
| GO:0071356 | cellular response to tumor necrosis factor | 0.045154 |
| GO:0051271 | negative regulation of cellular component movement | 0.045338 |
| GO:0009410 | response to xenobiotic stimulus | 0.045707 |
| GO:0010226 | response to lithium ion | 0.045788 |
| GO:0035809 | regulation of urine volume | 0.045788 |
| GO:0042094 | interleukin-2 biosynthetic process | 0.045788 |
| GO:0071379 | cellular response to prostaglandin stimulus | 0.045788 |
| GO:0072001 | renal system development | 0.045954 |
| GO:0010812 | negative regulation of cell-substrate adhesion | 0.045954 |
| GO:0019915 | lipid storage | 0.045954 |
| GO:1903317 | regulation of protein maturation | 0.045954 |
| GO:0050670 | regulation of lymphocyte proliferation | 0.046101 |
| GO:0032944 | regulation of mononuclear cell proliferation | 0.046927 |
| GO:0055076 | transition metal ion homeostasis | 0.04738 |
| GO:0015909 | long-chain fatty acid transport | 0.04738 |
| GO:0050766 | positive regulation of phagocytosis | 0.04738 |
| GO:0050707 | regulation of cytokine secretion | 0.047485 |
| GO:0032355 | response to estradiol | 0.048283 |
| GO:0044843 | cell cycle G1/S phase transition | 0.04834 |
| GO:0051043 | regulation of membrane protein ectodomain proteolysis | 0.04834 |
| GO:0072215 | regulation of metanephros development | 0.04834 |
| GO:0090140 | regulation of mitochondrial fission | 0.04834 |
| GO:1901032 | negative regulation of response to reactive oxygen species | 0.04834 |
| GO:1903206 | negative regulation of hydrogen peroxide-induced cell death | 0.04834 |
| GO:0051966 | regulation of synaptic transmission, glutamatergic | 0.048451 |
| GO:0001889 | liver development | 0.048787 |
| GO:0007292 | female gamete generation | 0.049951 |
| GO:0035976 | transcription factor AP-1 complex | 0.040968 |
| GO:0035259 | glucocorticoid receptor binding | 0.000861 |
| GO:0001228 | DNA-binding transcription activator activity, RNA polymerase II-specific | 0.001638 |
| GO:0001216 | DNA-binding transcription activator activity | 0.001638 |
| GO:0051427 | hormone receptor binding | 0.001638 |
| GO:0019955 | cytokine binding | 0.010034 |
| GO:0005124 | scavenger receptor binding | 0.013482 |
| GO:0035258 | steroid hormone receptor binding | 0.013482 |
| GO:0035257 | nuclear hormone receptor binding | 0.015749 |
| GO:0004982 | N-formyl peptide receptor activity | 0.015749 |
| GO:0016922 | nuclear receptor binding | 0.021554 |
| GO:0140297 | DNA-binding transcription factor binding | 0.04723 |
| GO:0061629 | RNA polymerase II-specific DNA-binding transcription factor binding | 0.047608 |
| GO:0001664 | G protein-coupled receptor binding | 0.047608 |
| GO:0005126 | cytokine receptor binding | 0.049874 |

**Supplement Table 2 GSEA enrichment analysis of genes co-regulated in both disease**

| **Description** | **NES** | **p.adjust** |
| --- | --- | --- |
| PATIL_LIVER_CANCER | 2.810268 | 6.80E-05 |
| KINSEY_TARGETS_OF_EWSR1_FLII_FUSION_UP | 2.338067 | 0.02525 |
| CREBP1_Q2 | -1.93395 | 0.049827 |
| BURTON_ADIPOGENESIS_PEAK_AT_2HR | -1.9407 | 0.047569 |
| GO_CELL_CHEMOTAXIS | -1.97159 | 0.044024 |
| GO_CELLULAR_RESPONSE_TO_OXYGEN_CONTAINING_COMPOUND | -2.00494 | 0.047569 |
| GO_RESPONSE_TO_STEROID_HORMONE | -2.00685 | 0.047569 |
| GO_REGULATION_OF_IMMUNE_SYSTEM_PROCESS | -2.00698 | 0.04406 |
| GSE23925_LIGHT_ZONE_VS_NAIVE_BCELL_UP | -2.01089 | 0.04171 |
| GSE37605_TREG_VS_TCONV_NOD_FOXP3_FUSION_GFP_UP | -2.01904 | 0.044024 |
| GO_SECRETION | -2.03315 | 0.042233 |
| GSE45365_NK_CELL_VS_BCELL_UP | -2.04028 | 0.047569 |
| REACTOME_CYTOKINE_SIGNALING_IN_IMMUNE_SYSTEM | -2.04507 | 0.047569 |
| GSE37605_FOXP3_FUSION_GFP_VS_IRES_GFP_TREG_C57BL6_UP | -2.06001 | 0.04171 |
| GO_POSITIVE_REGULATION_OF_SIGNALING | -2.08716 | 0.044024 |
| GSE34392_ST2_KO_VS_WT_DAY8_LCMV_EFFECTOR_CD8_TCELL_DN | -2.09008 | 0.044024 |
| GO_REGULATION_OF_MAPK_CASCADE | -2.10831 | 0.04171 |
| GSE27434_WT_VS_DNMT1_KO_TREG_DN | -2.12671 | 0.03496 |
| GO_POSITIVE_REGULATION_OF_GENE_EXPRESSION | -2.12679 | 0.031754 |
| NAGASHIMA_EGF_SIGNALING_UP | -2.14499 | 0.035964 |
| GO_POSITIVE_REGULATION_OF_CYTOKINE_PRODUCTION | -2.15043 | 0.029074 |
| GO_CYTOKINE_PRODUCTION | -2.1556 | 0.03496 |
| GO_DEFENSE_RESPONSE | -2.15686 | 0.029385 |
| GO_RESPONSE_TO_ORGANIC_CYCLIC_COMPOUND | -2.18089 | 0.029385 |
| ACEVEDO_LIVER_CANCER_DN | -2.19564 | 0.029385 |
| GSE46606_UNSTIM_VS_CD40L_IL2_IL5_1DAY_STIMULATED_IRF4HIGH_SORTED_BCELL_DN | -2.19923 | 0.029074 |
| GO_POSITIVE_REGULATION_OF_MULTICELLULAR_ORGANISMAL_PROCESS | -2.20844 | 0.031754 |
| GSE9988_ANTI_TREM1_VS_CTRL_TREATED_MONOCYTES_UP | -2.22868 | 0.029074 |
| HALLMARK_TNFA_SIGNALING_VIA_NFKB | -2.23146 | 0.029385 |
| GO_SIGNAL_TRANSDUCTION_BY_PROTEIN_PHOSPHORYLATION | -2.23879 | 0.029074 |
| GO_LOCOMOTION | -2.24942 | 0.02525 |
| ZWANG_CLASS_3_TRANSIENTLY_INDUCED_BY_EGF | -2.31272 | 0.02525 |
| GO_TAXIS | -2.49997 | 0.008669 |
